# On‐Chip Neural Induction Boosts Neural Stem Cell Commitment: Toward a Pipeline for iPSC‐Based Therapies

**DOI:** 10.1002/advs.202401859

**Published:** 2024-04-24

**Authors:** Saumey Jain, Dimitrios Voulgaris, Surangrat Thongkorn, Rick Hesen, Alice Hägg, Mohsen Moslem, Anna Falk, Anna Herland

**Affiliations:** ^1^ Division of Micro and Nanosystems KTH Royal Institute of Technology Malvinas väg 10 Stockholm 100 44 Sweden; ^2^ Division of Nanobiotechnology Science for Life Laboratory KTH Royal Institute of Technology Tomtebodavägen 23a Solna 171 65 Sweden; ^3^ AIMES Center for Integrated Medical and Engineering Science Department of Neuroscience Karolinska Institutet Solna 171 65 Sweden; ^4^ Chulalongkorn Autism Research and Innovation Center of Excellence (Chula ACE) Department of Clinical Chemistry Faculty of Allied Health Sciences Chulalongkorn University Bangkok 10330 Thailand; ^5^ Neural Stem Cells Department of Experimental Medical Science Lund Stem Cell Center Lund University Lund 221 84 Sweden; ^6^ Department of Neuroscience Karolinska Institutet Solna 171 65 Sweden

**Keywords:** differentiation, iPSC, microfluidic chip, microfluidics, neural stem cells, reprogramming, stem cell therapy

## Abstract

The clinical translation of induced pluripotent stem cells (iPSCs) holds great potential for personalized therapeutics. However, one of the main obstacles is that the current workflow to generate iPSCs is expensive, time‐consuming, and requires standardization. A simplified and cost‐effective microfluidic approach is presented for reprogramming fibroblasts into iPSCs and their subsequent differentiation into neural stem cells (NSCs). This method exploits microphysiological technology, providing a 100‐fold reduction in reagents for reprogramming and a ninefold reduction in number of input cells. The iPSCs generated from microfluidic reprogramming of fibroblasts show upregulation of pluripotency markers and downregulation of fibroblast markers, on par with those reprogrammed in standard well‐conditions. The NSCs differentiated in microfluidic chips show upregulation of neuroectodermal markers (*ZIC1*, *PAX6, SOX1)*, highlighting their propensity for nervous system development. Cells obtained on conventional well plates and microfluidic chips are compared for reprogramming and neural induction by bulk RNA sequencing. Pathway enrichment analysis of NSCs from chip showed neural stem cell development enrichment and boosted commitment to neural stem cell lineage in initial phases of neural induction, attributed to a confined environment in a microfluidic chip. This method provides a cost‐effective pipeline to reprogram and differentiate iPSCs for therapeutics compliant with current good manufacturing practices.

## Graphical abstract

This study highlights the development of a microfluidic platform to reprogram somatic cells from donors into induced pluripotent stem cells and further differentiate them into neural stem cells. This confined microfluidic platform boosts neural stem cell generation commitment at an early stage, as denoted by the pathway enrichment analysis.

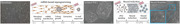

## 1 Introduction

The revolutionary discovery of reprogramming factors by Yamanaka et al. to revert somatic cells to their pluripotent stem cell state opened new avenues, especially in cell biology, diagnostics, and therapeutics, including patient‐specific disease modeling, testing of novel therapeutic modalities, and personalized genetic and therapeutic approaches.^[^ ^1^, ^2^, ^3^ ^]^ These reprogrammed cells, commonly referred to as iPSCs, are capable of self‐renewal and are pluripotent, that is, can be differentiated into any germ layer of interest. Non‐integrative reprogramming can show higher reprogramming efficiencies while bypassing virus‐induced genomic integration, thereby increasing the safety of such cell‐based therapeutics compared to the lentiviral induction methods.^[^ ^4^ ^]^ The development of non‐integrative reprogramming methods, such as synthetic and modified mRNA, has increased the interest in iPSC‐derived cell therapies.^[^ ^5^ ^]^

The clinical application and approval of such iPSC‐based therapeutics encounter obstacles due to the inherent variability of the cells. This variability hinders standardization, resulting in increased risks and difficulty in delivering a robust product.^[^ ^6^ ^]^ Most current pluripotent stem cell‐derived therapies are allogenic, that is, not derived from the patient that will be treated, and are under development at centralized production facilities. Large‐scale and robust production in these centralized facilities often results in high costs and a large amount of manual workload.^[^ ^7^, ^8^ ^]^ A critical drawback with these allogeneic therapies is that they require the patients to be on a long‐term immunosuppressive drug regimen, limiting the potential of such therapies and adversely affecting the patient's quality of life.^[^ ^7^, ^8^ ^]^ In addition, achieving large‐scale and robust production in these centralized facilities poses a challenge, often resulting in high costs and manual workload. The alternative, autologous cell therapies—involve reprogramming and differentiation of the patient's own cells and are in less demand of immunosuppressive treatment.^[^ ^9^, ^10^ ^]^ However, these are considered too costly and not easily accessible due to the prohibitive costs involved with the current reprogramming methods. To make such autologous cell therapies accessible and affordable, an efficient and standardized pipeline is necessary to enable sustainable reprogramming of somatic cells from a patient and further differentiate them into the required therapeutic cell type.

Currently, such cell therapy products are performed using standard systems such as well plates and flasks, enabling easy manipulation and regulation of the culture microenvironment. However, these culture methods cannot accurately control the cellular microenvironment and cell‐to‐cell signaling due to their large surface area and volume.^[^ ^11^, ^12^, ^13^ ^]^ On the contrary, dedicated microfluidic solutions and developments in microfabrication, surface chemistry, and molecular biology enable the precise control of the cellular microenvironment by reducing the culture volume, making it easier to handle, improving cost‐effectiveness, and having the potential for automation and parallelization.^[^ ^14^ ^]^ These can be easily integrated into downstream analysis and quality control methods such as imaging and qPCR.^[^ ^15^ ^]^ Previous works have reported the differentiation of human embryonic stem cell (hESC) derived embryoid bodies and highlighted that the gene and protein expression is regulated by biophysical parameters such as confinement, 3D microenvironment, and stress, which influence cellular proliferation and differentiation.^[^ ^16^, ^17^ ^]^ The inter‐cellular factors and signaling molecules are concentrated in a confined chamber, thus enhancing cellular uptake.^[^ ^18^ ^]^ Thanks to the low volume to surface area, microfluidic cell culture can form a cell culture environment that, unlike traditional cultures, allows the extracellular accumulation of signaling molecules and factors released in the media by cells undergoing reprogramming or differentiation. These concentrated signaling molecules act as cues for the other surrounding cells to initiate cellular processes like proliferation and differentiation. This results in a more homogenous cellular population as an increased concentration of endogenous signaling molecules drives specific cell fate decisions.^[^ ^19^, ^20^ ^]^ The initial protocols for microfluidic reprogramming of human somatic cells (dermal fibroblasts) into iPSCs were first reported and optimized by the group led by Elvassore in 2015.^[^ ^21^, ^22^, ^23^ ^]^ Further work in this regard also highlighted an approach for maintaining and inducing the three germ layers in a microfluidic platform from hESC and iPSC cultures but could not derive a functional ectodermal cell type.^[^ ^21^, ^24^ ^]^ Recent works involving culturing neural cells in a microfluidic setting usually require cells to be cultured in conventional well plates before being seeded into microfluidic devices for drug testing and disease model experiments.^[^ ^25^, ^26^, ^27^ ^]^ On‐chip generation of NSCs could be used in the future to develop a microfluidic pipeline to investigate their potential for use in neural cell therapies, for example, for neurodegenerative diseases such as Parkinson's disease, spinal cord injury, and stroke.^[^ ^10^, ^28^, ^29^, ^30^, ^31^, ^32^, ^33^ ^]^

**Figure 1:**
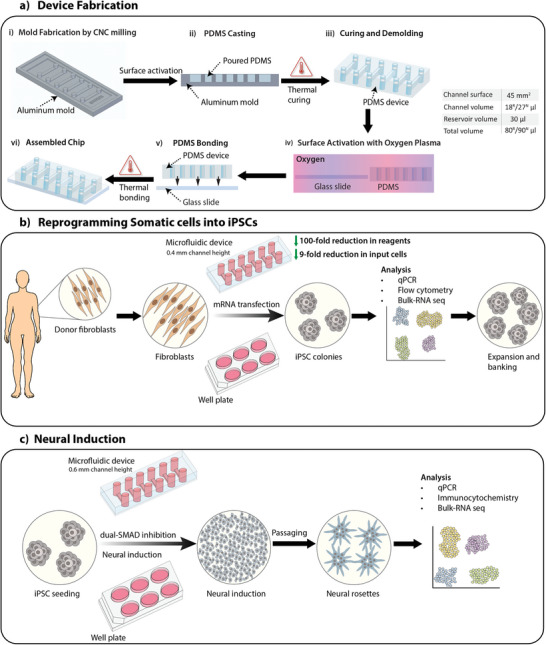
Overview of the study, highlighting the device fabrication, reprogramming of somatic cells into iPSCs, and neural induction of iPSCs using dual‐SMAD inhibition protocol to yield neural stem cells. a) Process flow for fabrication of the microfluidic device with 0.4 and 0.6 mm high channels used for somatic cell reprogramming (R) and neural induction (N), respectively, resulting in differences in channel volume and total volume indicated in table. b) Overview of the reprogramming process of somatic cells into iPSCs on both microfluidic devices and well plates using mRNA transfection. c) Overview of the neural induction process of iPSCs into neural stem cells on both microfluidic device and well plate using the dual‐SMAD inhibition protocol.

Here, we present a microfluidic chip platform for simplified and faster reprogramming of fibroblasts into iPSCs, followed by their in‐chip differentiation into NSCs (**Figure** **1**). This work advances and improves how iPSCs can be generated in microfluidic devices and demonstrates the first case of microfluidic generation of functional ectodermal cells toward the neural lineage as neural stem cells using the well‐known dual‐SMAD inhibition protocol. Our protocol results in reprogrammed iPSC colonies in about 10 days and NSCs in another 12 days. The proposed microfluidic pipeline includes chips that are easy to fabricate using PDMS and reduce the cellular input, reagent requirement, and manual labor, leading to substantial cost savings. The platform can be easily modified to enable adaptability for differentiation into other cell types. This is also a significant improvement over previously reported works wherein the expansion and differentiation were performed in the conventional well plate format using a high concentration of factors, partially negating the benefits of miniaturization in a microfluidic platform.^[^ ^21^, ^22^, ^23^ ^]^ We demonstrated that the chip‐reprogrammed and differentiated cell population had similar mRNA expression profiles to those in the conventional well‐plate format. We highlight the differences induced in a microfluidic system compared to a conventional cell culture platform using bulk RNA sequencing to study their clinical applicability and substantiate different biological functions and pathways in each culture format.

## 2 Results and Discussion

### 2.1 Design and Fabrication of the Microfluidic Device

We developed a microfluidic polydimethylsiloxane (PDMS) device platform with dimensions of 75 mm x 25 mm, matching the footprint of a standard microscope glass slide (Figure 1a; see Experimental Section for detailed fabrication process). Each microfluidic device consists of six channels with a height of 0.4 and 0.6 mm for reprogramming and neural differentiation, respectively. Each channel had a surface area of 45 mm^2^. The dimensions of the channels, the surface area, and the volume are described in Table S1 (Supporting Information). This platform was customizable and adaptable depending on cell and process‐specific requirements, as defined below. These devices had a substantially higher surface area (45 mm^2^) to volume (18 or 27 µL) ratio as compared to the conventional 6‐well (960 mm^2^ and 2000 µL) and 12‐well plates (350 mm^2^ and 1000 µL) used for cellular reprogramming and differentiation, respectively. Therefore, we achieved a 100‐ and 16‐fold reduction in reagent volumes used for reprogramming (channel height 0.4 mm) and differentiation (channel height 0.6 mm) compared to 6‐ and 12‐well plates. Compared to previous reports that used photolithography for pattern‐definition of the master molds, our mold designs were initially prototyped using a fast commercial 3D printer to iterate design specifications, followed by industry‐standard CNC‐milling to implement an adaptable and scalable chip production method (see Experimental Section).^[^ ^22^, ^23^ ^]^ The same articles reported an approximately 60‐fold reduction in mRNA and reprogramming reagents needed for reprogramming. The devices also permit easy media exchange (18 µL/27 µL volume to replace all media). Our design facilitated pipetting to chip interfaces and minimized flow‐induced shear forces. The simple interface allows for further automation, potentially as a closed system that can be used to develop personalized cell‐based therapeutics.

### 2.2 Microfluidic Reprogramming of Somatic Fibroblast Cells

**Figure 2:**
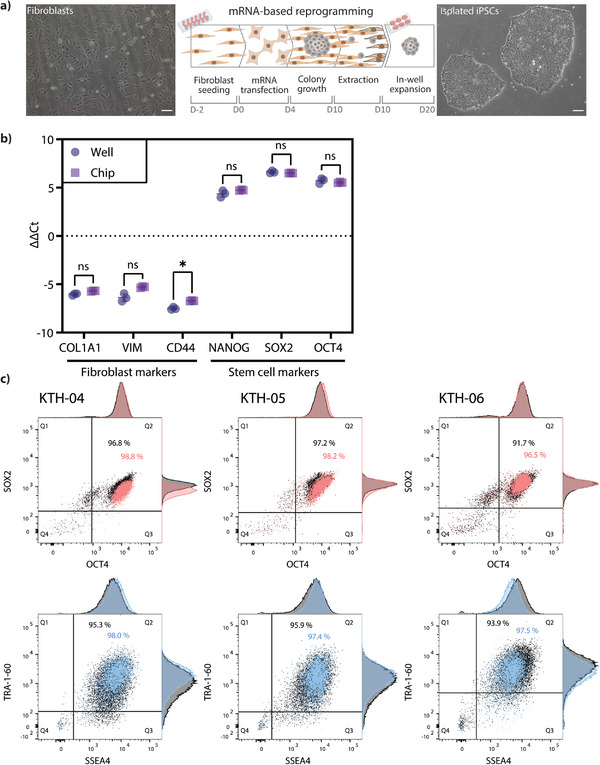
Reprogramming of fibroblasts into induced pluripotent stem cells. a) Illustrative protocol employed for the reprogramming of fibroblasts and bulk expansion of isolated colonies. Scale bar 50 µm. b) qPCR analysis of the reprogrammed iPSCs shows downregulation of mesenchymal markers and upregulation of pluripotency markers in both well and chip formats. c) Flow cytometry analysis for standard intracellular pluripotency markers *OCT4* and *SOX2* and extracellular pluripotency markers SSEA4 and TRA‐1‐60 in iPSCs reprogrammed in wells (black) or chip (red intracellular, blue extracellular). Lines delineate quadrants (Q1–Q4) of negative and positive signals for the two fluorophores, and the numbers in Q2 indicate the percentage of cells that are double positive for both markers. Data generated after single cell expansion. The gating strategy is shown in Figure S4 (Supporting Information). Panel (c) used the following cell lines KTH‐04: well p 19, chip p 20. KTH‐05: well p 14, chip p14. KTH‐06: well p 14, chip p 14.

To test our microfluidic platform initially, we used neonatal fibroblast cells and transfected them for four days. Upon colony growth and identification (**Figure** **2**a), iPSCs were removed by Versene treatment and expanded for downstream analysis. Compared to manual colony picking, this novel isolation method for iPSC colonies does not require manual cutting of the channels. Hence, unlike the manual colony‐picking method, this Versene‐based method could be implemented in automated settings. Since iPSCs are known to be less adherent than fibroblasts, the use of an enzyme‐free method selectively dissociates iPSCs from a mixed population of cells, effectively removing the need for a trained professional to perform the clonal selection.^[^ ^34^ ^]^ Isolated cells were passaged upon reaching 80% confluency on LN‐521 coated plates with the addition of Y‐27632. The cells formed colonies upon removing Y‐27632 from the media, a well‐known behavior while culturing stem cells in‐vitro.^[^ ^35^ ^]^ The iPSCs were expanded in the iPSC media during subsequent passaging. The iPSC lines established using this method are a non‐clonal expansion of a bulk culture.

Adult fibroblast cells are an ideal cell type to test the capabilities of microfluidic platforms, as it is challenging to culture cells in confined environments.^[^ ^22^ ^]^ These are ideal candidates as primary fibroblasts are easily available and minimally invasive sources of cells in a practical scenario.^[^ ^36^, ^37^ ^]^ Furthermore, the easy commercial availability of such cells makes them ideal cell sources for prototyping. To further assess our microfluidic platform, we performed side‐by‐side reprogramming using mRNA in a conventional well plate and microfluidic platforms using 3 different donors of fibroblast cells that varied in age, sex, and pigmentation (Table S2, Supporting Information). We achieved a 100% success rate in generating colonies in both well and chip culture conditions. In our protocol, following the transfection, more colonies appeared in the well conditions for two of the fibroblast cell lines used, the neonatal (KTH‐04) and one adult (KTH‐05), whereas the oldest fibroblast cell line had a low number of colonies for analysis (KTH‐06) in any of the culture conditions, which is attributed to the phenotypic, genetic and proliferative differences of the starting fibroblasts.^[^ ^38^, ^39^, ^40^, ^41^ ^]^ Specifically, neonatal fibroblast cells had 38 colonies/10 mm^2^ on average in the well conditions, whereas chip conditions had 20 colonies/10 mm^2^ on average (Figure S1, Supporting Information; well vs chip, p<0.0001). Adult fibroblast cells had, on average, 4 times fewer colonies compared to neonatal, 10 colonies/10 mm^2^. As with the neonatal ones, adult fibroblast cells reprogrammed in chips resulted in fewer colonies, 4 colonies/10 mm^2^ (Figure S1, Supporting Information; well vs chip, p = 0.0226). Notably, due to a lower surface area, the chips require ninefold fewer cells than the seeding in wells, even when similar seeding densities are used. Furthermore, the downscaling in the microfluidic chip leads to a 100‐fold reduction in reagents needed. The cells seeded in the well and chip were imaged daily to ensure they looked healthy and showed no signs of cell death.

After extraction and expansion, we further evaluated if there were any differences in expression levels of fibroblast markers and markers for undifferentiated stem cells related to pluripotency between the two culture conditions. We verified that the levels of the pluripotency markers were not significantly different between well and chip conditions (Figure 2b). The cells were characterized by qPCR at passages 6–8 to analyze the gene expression of reprogrammed cells relative to the parental fibroblast cells used for each specific reprogramming run performed in both well and chip formats in parallel. We validated the shift in the cellular state by a decrease in the relative expression of fibroblast‐specific genes, namely *COL1A1*, *VIM*, and *CD44* (Figure 2b). In contrast, we observed significantly enriched pluripotency‐associated genes *SOX2*, *NANOG*, and *OCT4* (Figure 2b) compared to the starting cell lines. Monitoring the undifferentiated and pluripotent status of iPSCs is essential for quality controlling the reprogramming process and the iPSC lines. Additionally, for preserving experiment quality and reproducibility, flow cytometry was used to quality control iPSC lines pluripotency status at later passages (KTH‐04: well p 19, chip p 20. KTH‐05 and KTH‐06: well p 14, chip p 14) All iPSC lines showed high expressions of the well‐defined and commonly used markers for pluripotency *OCT4*, *SOX2*, SSEA4, and TRA‐1‐60 (Figure 2c; Figures S2 and S3, Supporting Information).^[^ ^42^ ^]^ *OCT4* and *SOX2*, together with *NANOG*, belong to the pluripotency core regulatory network driving the identity of iPSCs.^[^ ^43^ ^]^ SSEA4 and TRA‐1‐60 are the two key extracellular pluripotent stem cell markers for iPSCs.^[^ ^44^, ^45^ ^]^ Expression profiles are consistent with pluripotency‐associated protein expression levels in established clonal iPSC lines obtained from the iPS Core at Karolinska Institute: Control 7‐II, Control 10‐V, and Control 14‐II, which were analyzed using the same multicolor flow cytometry panel and instrument settings (Figure S4, Supporting Information).In addition, flow cytometry analysis of intracellular and extracellular markers confirms that well and chip conditions have very similar pluripotency‐related expression, with 91.7%–98.8% cells double positive for OCT4 and SOX2 and 93.9%–98.0% double positive for SSEA4 and TRA‐1‐60 (Figure S5, Supporting Information).

To further assess the comparability of our generated iPSCs, we ventured into bulk RNAseq analysis to explore differences on a global scale. To compare the generated lines with known standard, we included three iPSC lines obtained from the iPS Core at Karolinska Institutet as a well‐established reference data set (Table S4, Supporting Information).^[^ ^46^, ^47^ ^]^ The bulk RNA sequencing data was analyzed using the protocol listed in the Experimental Methods section. We assessed the quality of our generated lines (reprogrammed through conventional wells and microfluidic chips, KTH‐04, ‐05, and ‐06) by comparing them to the reference lines (Table S4, Supporting Information). We observed that each cell line clusters close to each other and the reference lines (**Figure** **4**). Further strengthening the validation of our proposal system, Principal Component Analysis (PCA) revealed that the reprogrammed cells, both in well and chip format arising from the same fibroblast cell line, cluster together (Figure S6, Supporting Information), showing no significant differences between the iPSCs derived from the same cell source. There is, however, some difference between the iPSCs derived from different cell sources, which is a known effect arising due to the genetic variability, epigenetic factors, and the choice of the reprogramming method used.^[^ ^39^, ^48^, ^49^ ^]^ Although there are minor differences in the PCA plot, the biological variability between the reference lines is much higher than the differences between well and chip conditions per reprogrammed iPSC line.^[^ ^19^, ^39^ ^]^

Some previous works have reported an increased number of colonies per unit surface area in the chips compared to the well plates.^[^ ^22^, ^23^ ^]^ In our system, we observed more efficient reprogramming, especially considering the cumulative amount of mRNA transfected over 4 days of mRNA transfection per number of input cells, making the process simple, cost‐efficient, and straightforward. We did not observe more colonies per area in the chip format, and we attribute this to the starting donor fibroblasts used. As we observed and has been previously reported, donor‐related factors like differences in age, gender, pigmentation, and other factors have a marked effect on reprogramming, affecting the efficiency and the resulting cells.^[^ ^50^, ^51^, ^52^ ^]^ Nevertheless, any pipeline for stem cell therapies would require the banking of chip‐generated iPSCs; thus, expansion outside the chip is needed. Hence, as long as each chip reprogramming results in proliferative, healthy iPS cells, as our method does, a lower efficiency in the number of colonies per area does not impede any downstream processes or analysis. Like all cells, iPSCs interact with the cell culture medium around the cells through regular release and uptake of molecules through the cell membrane. Conventional cell culture methods have a high media volume to cell number ratio, significantly diluting the secreted factors in the media and limiting autocrine and paracrine signaling.^[^ ^53^ ^]^ The mixing of reagents and cell‐secreted compounds in simple microfluidic devices and conventional well plates is passive, making the mass transport of components limited by diffusion.^[^ ^54^, ^55^ ^]^ According to Fick's law of diffusion, the diffusion time is proportional to the square of the distance in a passive system with diffusion limitation.^[^ ^56^, ^57^ ^]^ In the case of a conventional well plate (≈8 mm), the average distance traveled is much longer than in the microfluidic chips (0.4 mm), making it up to 25 times faster to get an even distribution of a generic compound in the chip.

### 2.3 Microfluidic Differentiation into Neural Stem Cells

#### 2.3.1 Differentiation of iPSCs into NSCs

**Figure 3:**
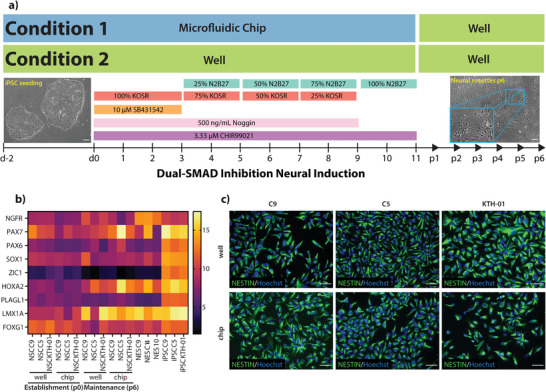
Comparing neural differentiation in well and chip culture conditions. a) Experimental conditions implemented for neural induction on‐chip (Condition 1) and compared to the well plate conditions (Condition 2). The neural induction protocol ends at d11, and the part after d11 represents the expansion and maintenance of the NSCs. b) qPCR analysis (ΔΔCt values) of neural markers in differentiated cells in well‐ and microfluidic formats. The NES cell lines are used as a reference. iPSC C9, iPSC C5, and iPSC KTH‐01 are the starting lines differentiated into neural stem cells. c) ICC images of NESTIN for NSC C9, C5, and KTH‐01 generated in chips and wells. Scale bar 50 µm.

To further evaluate possibilities to control cell states in microfluidic culture, we evaluated if we could differentiate iPSCs into neural lineages in the chip. The neural lineage specification is particularly interesting in microfluidic format as it is heavily dependent on cell‐to‐cell signaling. However, previous reports on microfluidic differentiation demonstrated functional endodermal and mesodermal lineages but not ectodermal.^[^ ^21^, ^33^, ^55^, ^58^, ^59^ ^]^ We took one of the most well‐known protocols to generate NSCs, the dual‐SMAD inhibition protocol, and optimized our chip design to suit the protocol.^[^ ^60^, ^61^ ^]^ To the best of our knowledge, this is the first report demonstrating the downscaling of this neural induction protocol beyond the conventional 2D cell culture formats used. The dual‐SMAD inhibition protocol targets the Bone Morphogenetic Protein (BMP) and the Transforming growth factor‐β (TGF‐β) pathways using Noggin and SB431542.^[^ ^60^, ^62^, ^63^ ^]^ In our optimization of chip parameters, we found that the chips with 0.6 mm height resulted in excellent cell survival over passages, whereas 0.4 mm was found to be sub‐optimal. We attribute this need for a higher height and, thus, chip volume to the demand for high cell density to achieve an efficient induction into neural stem cells.^[^ ^60^, ^64^ ^]^ A high cell density requires more nutrients and, thus, a larger media volume per area. We compared neural induction performed in conventional wells and chips side‐by‐side with daily media and induction reagent replenishments as depicted in **Figure** **3**a and explained in detail in Experimental Section and Tables S5 and S6 (Supporting Information).

**Figure 4:**
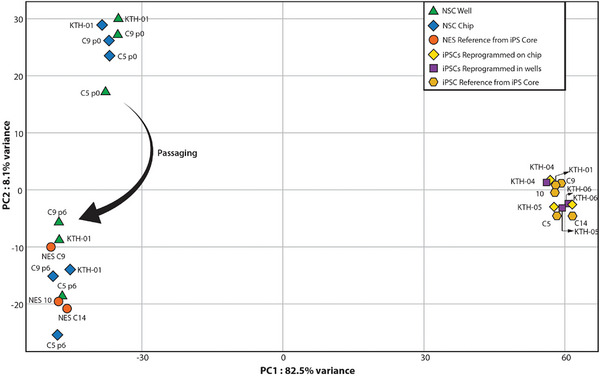
PCA of NSCs obtained by differentiation of iPSCs in microfluidic and conventional well plate format. NES cells are used as references. There is a transcriptomic shift of NSCs toward NES cell lines after six passages.

To characterize the NSC generation, we established two points for this analysis, namely, generation and maintenance. Generation reflects the time point precisely at the end of the neural induction protocol (passage 0 or p0, day 11 of the protocol). In contrast, maintenance reflects the time after cells were passaged 5–6 times outside the chip (passage 6 or p6). During that time, the culturing format we followed was according to Figure 3a. We also included three neural stem cell lines (termed NES) previously established as a reference in our analysis (Table S4, Supporting Information; first shown by Falk et al.).^[^ ^64^ ^]^ Importantly, one of the iPSC (KTH‐01) lines subjected to neural induction in chip and well was generated with chip reprogramming and thus served as an additional quality control of our reprogramming method. We assessed neuroectoderm‐ and neural stem cell‐associated markers initially by qPCR. Regardless of the culturing system (chip or well) or the time points (p0 or p6), all lines showed enrichment in neural progenitor genes such as *ZIC1*, *SOX1, PAX6*, and neural rosette marker *PLAGL1* (Figure 3b) compared to the iPSC state as is expected for NSCs.^[^ ^60^, ^64^ ^]^ From the genes investigated in qPCR, only *LMX1A* was downregulated between NSC generation and maintenance, standing out from the rest. Furthermore, we observe a higher expression of *FOXG1*, a forebrain regionality marker at the generation time point (p0), which decreases over time toward the maintenance time point (p6). This was accompanied by an enrichment of *HOXA2*, a hindbrain regionality marker from generation to maintenance, reaching levels comparable to the NES used as control. This is characteristic of neural stem cells undergoing posteriorization, transitioning from forebrain to hindbrain regionality.^[^ ^64^, ^65^, ^66^ ^]^ We also performed statistical analysis on the qPCR data in Figure 3b (Figure S7, Supporting Information). We observed a significant difference in *SOX1*, *PAX6*, *PLAGL1*, *FOXG1*, and *HOXA2* expression within the chip and well conditions at the NSC generation state. Similarly, at the NSC maintenance state, a significant difference was observed for PAX6, *HOXA2*, and *PLAGL1*. When we compared the gene expression of the NSCs obtained from both culturing conditions with the gold standard NES from the iPS Core Facility at Karolinska Institutet, we observed a significant difference in the expression of *ZIC1*, *PAX6*, and *PLAGL1*. Furthermore, the NSCs obtained from two culture conditions were found to be positive for Nestin, a neural stem/progenitor cell marker from the immunocytochemistry analysis (Figure 3c). As the qualitative immunocytochemistry analysis did not reveal any difference and we could not determine any downstream effects from the cells differentiated in well or chips from the qPCR, we decided to study the differences deeper with bulk‐RNA sequencing. While there was a noticeable transcriptomic difference observed in NSC between generation and maintenance, there was no noticeable difference between well and chip conditions in either of the time points, except NSC CTRL‐05 (C5) standing out among the two other lines (maintenance, well, and chip conditions, Figure 4). PCA analysis of well conditions (generation and maintenance) indicates that NSC showed a change in their transcriptomic behavior over passaging from generation to maintenance, with the latter clustering closer together with the reference lines (Figure 4). This transcriptomic shift during the maintenance of cells generated through dual‐SMADi has been documented previously.^[^ ^67^ ^]^ The inherent variability between the lines is a product of cellular heterogeneity and differences inherent to the starting cell line, and it is well known that minute differences in the culture conditions can result in variations in gene expression and epigenetics of resulting cells.^[^ ^68^, ^69^, ^70^ ^]^

#### 2.3.2 Neural Induction on Chips Increases Neuralization of iPSCs

**Figure 5:**
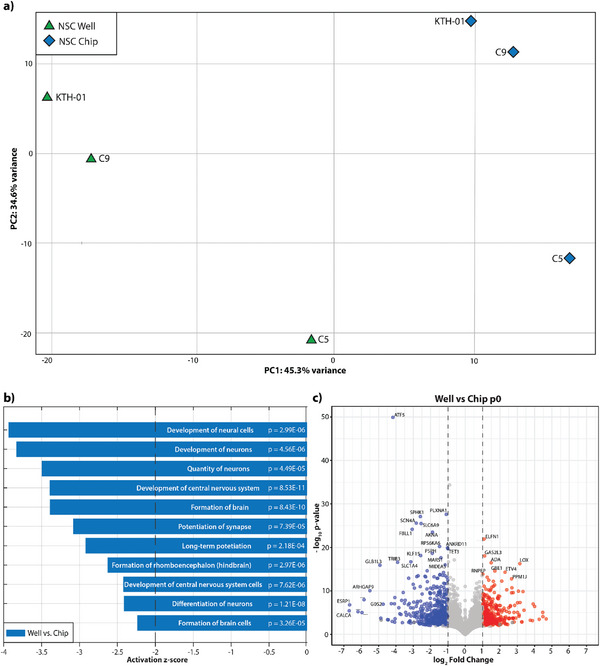
Neural differentiation at p0: a) PCA of NSCs obtained by differentiation of iPSCs in chips and well format at passage 0. It is observed that the cells differentiated in chips cluster closer to each other compared to those cultured in well plates. b) Biological functions identified and predicted to be upregulated in the chip at passage 0 compared to the chip using the downregulated genes in the wells. The cut‐off z‐score of −2 is set for the downregulation of functions. The p‐value for each biological function is calculated by IPA using Fisher's exact test. c) Volcano plot showing the differential expression analyzed by the culture format at passage 0 (well vs microfluidic chip) red and blue refer to the enrichment in chip and well conditions, respectively, whereas gray represents non‐significant genes.

**Figure 6:**
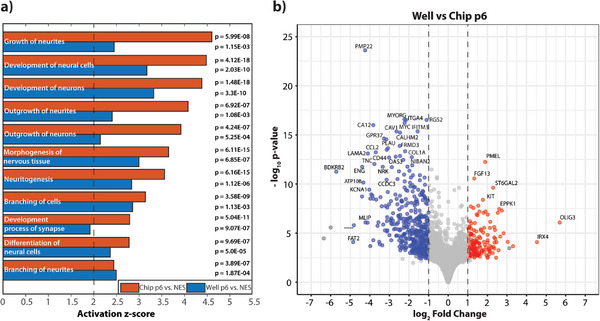
Neural Differentiation at p6: a) Common biological functions identified and predicted from the comparison analysis of the upregulated DEGs in chip/well compared to NES. The functions above the z‐score of 2 are predicted to be upregulated. The p‐value for each biological function is calculated by IPA using Fisher's exact test. b) Volcano plot showing the differential expression analyzed by the culture format at passage 6 (well vs microfluidic chip) red and blue refer to the enrichment in chip and well conditions, respectively, whereas gray represents non‐significant genes.

In analysis with PCA, notably along the PC1 axis, the timepoint “generation” revealed that cells clustered together per culture system, neural stem cells generated in chips clustered together with other lines generated in chips, and the same pattern followed for the neural stem cells generated in wells (**Figure** **5**a). However, as indicated by the RNAseq, C5 slightly deviated in well and chip conditions, where the line, regardless of the culturing system, did not cluster together with the other lines. Nevertheless, even in C5, there was a separation between well and chip conditions. Volcano plots from DEGs (Figures 5c and **6**b) showed that lines generated in chips had the most DEGs, suggesting that the culturing environment greatly influences cellular processes. Hierarchical clustering confirms the PCA plot (Figures S8 and S9, Supporting Information), where C9 and KTH‐01 NSC cluster in a chip versus well. Even though C5 did not cluster tightly together, pathway analysis of DEGs revealed that cellular development processes are highly enriched in NSC generated through chips (Figure 5b). Additionally, the DEGs are significantly upregulated, suggesting that those developmental processes are associated with neural system development when analyzed using Ingenuity Pathway Analysis. The differential expressed genes at P0 were obtained by filtering the dysregulated genes from RNA‐seq with log_2_ fold change less than −1 and more than 1 together with a p‐value < 0.05. We obtained 701 DEGs in the chip when compared to the well. We analyzed the DEGs using the Ingenuity Pathway Analysis to identify biological functions associated with cellular development and nervous system development. Further, we found 260 DEGs, which are predicted to be upregulated in chip culturing conditions compared to wells (negative z‐score, well vs chip). To remove the false discoveries, we only consider functions whose absolute activation score is above 2 (indicated by a dashed black line). At the timepoint “generation,” or P0, pathway analysis reveals that essential biological functions for Nervous System Development and Function, including “Development of neural cells” (p‐value = 2.99E‐06), “Quantity of neurons” (p‐value = 4.56E‐06), “Development of central nervous system” (p‐value = 8.53E‐11), “Formation of brain” (p‐value = 8.43E‐10), “Long‐term potentiation (p‐value = 2.18E‐04)” and “Formation of rhombencephalon” (p‐value = 2.97E‐06) among others were predicted to be upregulated in the cells differentiated in chips and downregulated in the ones differentiated and maintained in wells (Figure 5b). Out of these, “Formation of rhombencephalon” is particularly interesting as it signifies a distinct regional specification of the cells, which is characteristic of the NES‐like behavior previously reported in the literature.^[^ ^64^ ^]^ Furthermore, we observe that at p0, the molecular and cellular function ‘cell death and survival’ was found to be upregulated, an enhancement in 83 differentially expressed genes in the cell death and survival pathway in the cells neurally induced on the well as compared to the chip. This can indicate higher quality and efficiency in obtaining cells by conducting neural induction on the chip.

In the NSC timepoint “maintenance,” the PCA plot revealed a different pattern (Figures 4 and 5a). In time point “generation,” the cells clustered close to each other irrespective of the culturing system. While, in time point “maintenance,” there was no distinct clustering depending on the culturing system. Albeit chip conditions for C5 did not cluster closely with NSC CTRL‐09 (C9) and NSC KTH‐01 (chip conditions), it is still noteworthy that chip culturing conditions resulted in less spread of the cell lines. Moreover, NSC CTRL‐09 and NSC KTH‐01 on the chip were closer to the reference NES lines on the PC1 axis. We observed an increased spread between the lines in the well culture conditions. C5 did not cluster with the rest of the lines. We attribute this to the reprogramming technique used for the cells (Sendai virus for C5 vs mRNA for KTH‐01 and C9; Table S3, Supporting Information). It has been previously reported that variability of cell lines and differences in the culture conditions and protocols employed can lead to variation in gene expressions and epigenetic behavior.^[^ ^68^, ^69^ ^]^ Another difference in C5 compared to C9 and KTH‐01 is iPSC culture in defined conditions throughout all passages of the cell lines. This further exemplifies the need for standardized and controlled culture conditions. Nevertheless, it remains to be seen if and to what extent different reprogramming techniques affect the responsiveness of iPSCs to different culturing systems.^[^ ^17^, ^71^ ^]^

The differential expression of genes in chip and well culture conditions compared to the reference NES cells were obtained using similar statistical parameters as mentioned previously and in Experimental Section. We found 776 upregulated DEGs and 384 upregulated compared to the chip versus NES and well versus NES, respectively. These upregulated genes were uploaded to Ingenuity Pathway Analysis (IPA) software to predict the biological function and pathways. To remove the false discoveries, we only consider functions whose absolute activation score is above 2 (indicated by a dashed black line). We further observed the comparison between the biological pathways among these DEGs. At the timepoint “maintenance,” or p6, pathway analysis reveals that common biological functions for Nervous System Development and Function between the two comparisons showed higher activation scores for the chip versus NES, signifying that there is higher activation of upstream regulators in case of chips as compared to wells, especially for “Growth of neurites” (p‐value_chip_ = 5.99E‐08, p‐value_well_ = 1.15E‐03) and “Development of neural cells” (p‐value_chip_ = 4.12E‐18, p‐value_well_ = 2.03E‐10) (Figure 6a).

Stem cell differentiation and reprogramming can vary due to donor‐specific reasons and variability in cell culture conditions due to reagents and manual handling affecting the local milieu, as well as the inherent differences between the starting cell lines and minute differences in the culture conditions.^[^ ^68^, ^69^ ^]^ Mixing reagents and cell‐secreted compounds in simple microfluidic devices and conventional well plates is passive, limiting the mass transport of components by diffusion.^[^ ^56^ ^]^ As per Fick's law of diffusion, the diffusion of compounds in a system with diffusion limitation varies inversely with the square of the distance.^[^ ^54^ ^]^ The diffusion of molecules in a conventional well plate is significantly slowed (≈ 25‐fold slower) compared to the microfluidic chips because of the diffusion distance in the two scenarios (≈8 mm for wells vs. 0.4/0.6 mm for chips). Due to the high media volume, the endogenous and paracrine signaling factors cells release are diluted in the well plate culturing conditions.^[^ ^11^, ^57^ ^]^ As a result, their concentrations can be below thresholds of action. Moreover, culture can create concentration gradients, leading to inhomogeneous differentiation. However, in the case of microfluidic confinement, the dilution of cell‐released factors is not that drastic. In addition, diffusion across the whole channel occurs rapidly, leading to a more homogeneous culture environment.^[^ ^22^, ^72^ ^]^ Our data suggest that the microfluidic environment of the chip affects the milieu, as observed by the upregulation of terms associated with neural system development and function in chips compared to well at the generation time point (Figure 5b). This difference was also observed at the maintenance time point, where the cells differentiated in chip conditions had a higher activation score than those differentiated in well conditions (Figure 6a). We postulate that the confined environment in a microfluidic chip can reduce variations in the lines obtained, leading to an early neuronal fate specification compared to the conventional well format. Further, a microfluidic method requires less reagents, making it cost‐effective, and has the potential to be automated, providing a closed system with minimal variability to ensure a highly homogenous cell population as required by cGMP guidelines for use in therapeutics.^[^ ^12^, ^13^ ^]^

## 3 Conclusion

We present a straightforward and cost‐effective method to produce NSCs from somatic cells. The reprogramming step is done with mRNA transfection, utilizing a non‐integrative approach of generating stem cells compatible with therapeutics, as genomic integration is one of the significant limitations for developing therapeutic approaches. Our protocol, which showed a 100% success rate per technical replicate, is shorter than previous protocols (4 vs. 12 days previously reported) and consumes less reagents while showing minor differences in the colony formation between the reprogramming carried out in the well and microfluidic chips. Our method further allows the dissociation and isolation of iPSCs and NSCs without cutting open the microfluidic chip, highlighting the possibility of automation and minimal manual intervention. The cost‐effective approach of saved reagents in microfluidic chips and the yield of a standardized cell population overcome the marginally increased efficiency (colonies mm^−2^) observed in the conventional well‐culturing format. Although the number of reprogrammed cells obtained in a microfluidic device is smaller than in a conventional well plate, cell banking, and expansion make the method more sustainable, primarily since iPSCs can be readily expanded. Furthermore, the proposed microfluidic pipeline includes chips that are easy to fabricate and reduce the cellular input, reagent requirement, and manual labor, leading to substantial savings compared to prohibitive costs associated with reprogramming cells in a well format.

Additionally, microfluidic methods have the potential to be automated, providing a closed system with minimal batch‐to‐batch variability. Automation would thus ensure consistency, continuity, and reliability in producing highly homogenous cell populations, as required by cGMP guidelines for use in therapeutics. We have also shown a successful generation of neural stem cells from iPSCs in a microfluidic platform, with a boosted commitment to the neural fate at an earlier time point than those differentiated in a conventional well plate format. The significant upregulation of neural growth and differentiation pathways in cells differentiated on the microfluidic platform indicates a mature and uniform phenotype compared to those differentiated on the wells. Importantly, we documented that the confined environment of a microfluidic platform boosts neural stem cell generation commitment, as denoted by the pathway enrichment analysis. Stem cell differentiation is inherently variable and poses a problem for stem cell therapies. A microfluidic platform can reduce biological variability by providing an environment with cost‐efficient and standardized processes. In summation, the microfluidic chip platform holds the potential for the highly controlled generation of clinical grade iPSCs and differentiated cells for cellular therapeutics, potentially improving the transition of such therapy from the lab bench to the patient's bedside.

## 4 Experimental Section

### Device Fabrication

The aluminum master mold was designed using Siemens Solid Edge 2020 and milled using a CNC milling machine (Minitech Mini‐Mill/GX). The mold was cleaned with toluene and isopropanol and coated with 0.5% w/v Alconox (1104‐1, Alconox Inc.) in deionized water. The PDMS devices were fabricated using Sylgard 184 Elastomer (Base: Curing Agent; 10:1) and cured at 65 °C overnight. The PDMS was then demolded from the mold and washed and dried to remove any trace of Alconox. The PDMS devices were then bonded onto a glass slide using an oxygen plasma treatment to ensure a tight seal and bonding. The PDMS microfluidic devices were then cleaned using a UV‐ozone cleaner (Model 30, Jelight Company Inc.), followed by short‐term storage in 70% ethanol.

### Reprogramming

The PDMS devices fabricated per the method described above were coated with 10 µg mL^−1^ recombinant human laminin 521 (LN521, BioLamina AB) in DPBS+/+ (14040‐091, Thermo Fisher) by incubating the solution in the reservoir and the channels overnight. The solution was then aspirated out of the channel the next morning and washed with DPBS‐/‐ (14190‐094, Thermo Fisher), followed by a fibroblast medium consisting of Iscove's Modified Dulbecco's Medium (IMDM, 12440053, Thermo Fisher) with 10% fetal bovine serum (FBS, A3160801, Thermo Fisher) and 1% penicillin‐streptomycin (P/S, 15140122, Gibco). The primary fibroblasts were used as the starting cell line with a targeted seeding density of 75 cells mm^−2^ (300 cells mm^−2^ for the adult ones) in the devices using around 3500 neonatal fibroblasts or 13500 adult fibroblasts. 48 h after the fibroblast seeding, the media was aspirated from the reservoirs, and the channels were washed and replenished with the Essential 8 (E8) media (A1517001, Gibco). The mRNA for transfection was prepared using the instructions from the StemRNA 3rd Gen Reprogramming Kit (00‐0076, Stemgent). Four hour after the media change to E8, the media was aspirated from the reservoir using a pipette, and 16 µL of mRNA mix was introduced into the reservoirs using a pipette. The media was then circulated twice throughout the channel. The cells were maintained at 37 °C and 5% v/v CO_2_. The E8 washing and mRNA transfection were carried out for 3 days. In order to boost the reprogramming efficiency, Human iPS reprogramming booster supplement II (SCM094, EMD Millipore) was used alongside the StemRNA mix to improve and boost the reprogramming efficiency on day 2 and 3. The media was exchanged daily in the following days, and the chip was observed under a brightfield microscope to characterize and identify iPSC colony formation. The colonies could be identified from day 6 onward and were isolated after day 10. The isolation and harvest of the reprogrammed fibroblasts was carried out using pre‐warmed Versene (15040066, Thermo Fisher). EDTA‐containing solutions like Versene produce faster detachment in iPSCs than other types of cells, such as fibroblasts, as reported in the literature.^[^ ^34^ ^]^ This effect leads to preferential harvesting of iPSC colonies while most of the non‐reprogrammed fibroblasts remain attached. The isolated reprogrammed cells were expanded in E8 on a 6‐well plate coated with 5 µg mL^−1^ of LN 521in DPBS +/+, and passaging was done with Versene.

### Neural Induction

The devices with 0.6 mm channel height were used for neural differentiation of the reprogrammed cells. The channels were incubated with 50 µg mL^−1^ of LN521 to promote better cell adherence for 12 h in an incubator at 37 °C and 5% v/v CO_2_. The channels were washed with DPBS ‐/‐ to remove unbound LN521 and mTeSR1 (#85857, StemCell Technologies) + 1% P/S to equilibrate the channels to the culture conditions. The iPSCs were introduced at a seeding density of 400 cells mm^−2^ and supplemented with 10 µm Y‐27632 Rock inhibitor (SCM075, Sigma).

The neural induction media was prepared as described in Tables S5 and S6 (Supporting Information) and introduced into the channels by washing with the media in multiple steps. Meanwhile, a new microfluidic device was coated with 50 µg mL^−1^ of LN521 and incubated overnight to prepare for passaging on day 4. The media was aspirated, and the channels were washed with DPBS ‐/‐. TrypLE Select (12563‐029, Thermo Fisher) was then perfused into the channels and kept in the incubator for 5 min to detach the cells. The cell suspension was then collected by applying some shear stress by manual pipetting. The TrypLE Select was then deactivated by adding more media. The cells were spun down, and the pellet was resuspended in fresh media. The cells were then seeded into the devices at a seeding density of 1800 cells mm^−2^ with 10 µm Y‐27632 Rock inhibitor. The media was changed for the channels from day 6 to 9. On day 10, Noggin was removed from the media, and the devices were replenished with the media without Noggin. Depending on the sample, a 12‐well plate or a microfluidic device was prepared for further passaging by coating 100 µg ml^−1^ poly‐L‐ornithine (3655, Sigma Aldrich) in DPBS +/+ for 24 h at 37 °C, culture vessels were washed thoroughly with DPBS +/+, and then 1:100 solution of Laminin (Engelbreth‐Holm‐Swarm murine sarcoma basement membrane, L2020, Sigma Aldrich) in DPBS+/+ was added for 24 h at 37°C. The cells were dissociated using TrypLE Select, and the channels were washed with DPBS ‐/‐ to collect all the cells. TrypLE Select was deactivated by the addition of DMEM/F‐12 GlutaMAX (31331028, Gibco) in excess. The cells were then spun down, and the pellet was resuspended in the N2B27 propagation media (A1370701, 17504044, Gibco). The cell number was determined by manual counting using a hemocytometer. The cells were then seeded in a 12‐well plate at a seeding density of 10^6^ cells per well. The NSCs were then expanded and propagated in well plates.

### qPCR

RNA samples were prepared by lysing 500 000–2 000 000 cells per sample in Qiagen lysate buffer supplemented with β‐mercaptoethanol and stored at −80 ^○^C. RNA was isolated using the RNeasy mini kit (74104, Qiagen) following the company's instructions. The RNA concentration was determined using a nanodrop spectrometer (mySPEC, VWR). cDNA was synthesized with equal amounts of RNA per sample using the high‐capacity RNA‐to‐cDNA Kit in a thermal cycler (UNO96, VWR) at 37 ^○^C for 1 h, followed by 5 min at 95^○^C. The cDNA was added in triplicate per sample per Taqman gene expressions assay (listed in Table S7, Supporting Information), including GAPDH as a reference in each well in a 96‐well plate. The rt‐qPCR was initiated with a single cycle of 2 min at 95 ^○^C and 20 s at 60 ^○^C, after which a 40‐cycle PCR started with each cycle consisting of 3 s at 95 ^○^C, followed by 30 s at 60 ^○^C.

### ICC

Cells were seeded out at 40000 cm^−2^ and were fixed after 2–3 days with 4% paraformaldehyde (PFA, 10 min at room temperature). After washing twice with DPBS, cells were incubated with a blocking buffer (10% Goat serum (G9023, Sigma Aldrich) and 0.1% Triton‐X (HFH10, Thermo Fisher) in DPBS) for 1 h at room temperature. Then, cells were washed once with DPBS and incubated with NESTIN (MAB1259, R&D Systems, 100X) in dilution buffer (1% Goat serum and 0.01% Triton‐X in DPBS) overnight at 4 °C. Following the primary incubation, cells were washed and incubated with goat anti‐mouse 555 secondary antibody (SAB4600302, Sigma Aldrich, 500X in dilution buffer) for 1 h at room temperature. After that, cells were washed once and counterstained with Hoechst 33342 (H3570, Gibco, 2000X in dilution buffer). Finally, cells were washed twice with DPBS and imaged.

### Flow Cytometry

For iPSC characterization, cells were expanded in E8 in T25 flasks coated with 5 µg mL^−1^ of LN 521 in DPBS +/+. 10 µm Y‐27632 Rock inhibitor was added to the culture medium on the day of passage. Cells harvested using TrypLE Express were resuspended in DPBS to a concentration of 1 × 10^6^ cells mL^−1^ and stained with LIVE/DEAD Fixable Violet Stain (L34955, Thermo Fisher) according to the manufacturer's instructions. After washing with autoMACS Running Buffer (130‐091‐221, Miltenyi Biotec), cells were divided into separate Eppendorf tubes for extracellular staining. Cells 0.5 × 10^6^ cells in 100 µL autoMACS Running Buffer was used for each staining sample. Anti‐human PE‐conjugated TRA‐1‐60 (130‐122‐921, Miltenyi Biotec, 100X dilution) and PE‐Vio770‐conjugated SSEA4 (130‐128‐254, Miltenyi Biotec, 400X dilution) antibodies were added prior to 20 min incubation at 4 °C protected from light. Cells were washed twice in autoMACS Running Buffer before fixation and permeabilization using the Foxp3 Transcription Factor Staining Buffer Set (00‐5523‐00, Thermo Fisher) following the manufacturer's instructions. For intracellular staining, cells were suspended in 100 µL permeabilization buffer and stained with anti‐human APC‐conjugated OCT4 (130‐123‐257, Miltenyi Biotec, 200X dilution) and AF488‐conjugated SOX2 (560301, BD Biosciences, 40X dilution) antibodies for 20 min at 4 °C protected from light. After two washes with permeabilization buffer, cells were resuspended in 300 µL autoMACS Running Buffer and analyzed using BD LSR Fortessa X20. Data was evaluated with FlowJO software V10.8.2 (BD Biosciences). The proportion of pluripotency marker expression in fully stained samples was assessed by transferring gating from FMO controls with < 0.5% positive events.

### Bulk RNA Sequencing and Data Analysis

Total RNA was extracted from the cells using a Qiagen RNeasy Mini Kit. The initial RNA quality and concentrations were analyzed using Agilent Tapestation per the manufacturer's instructions. The libraries suitable for Illumina sequencing were prepared using Illumina Stranded mRNA prep ligation sample preparation protocol with a starting concentration of about 200 ng of total RNA. This protocol includes mRNA isolation, cDNA synthesis, adapter ligation, and subsequent amplification of the indexed libraries. The quality and yield of the amplified libraries were analyzed by fluorescence quantification using Qubit by ThermoFisher. The quality was checked using Agilent TapeStation. The indexed cDNA libraries were normalized and combined, followed by sequencing the pools on the Illumina NextSeq 2000 for a P2 100 cycle sequencing run that generated paired‐end reads with dual index. The base calling and demultiplexing of the paired‐end reads were performed using Illumina bcl2fastq (v2.20). The adapter trimming was done using BBMAP (v38.41). The sequence data quality was then assessed using FastQC (v0.11.8). The reads were then aligned to the Ensembl GRCh38 reference genome using STAR (v2.6.1d). The gene counts were estimated using featureCounts (v1.5.1). The count data was then imported into the Bioconductor package DESeq2 (V1.34) to test for differential expression using Wald tests.

### Pathway Enrichment Analysis

The prediction of neural system development of differentially expressed genes were analyzed using Ingenuity Pathway Analysis (IPA) (QIAGEN Inc.,). The list of differentially expressed genes was overlapped with the list of genes experimentally validated to be associated with neural system development in Ingenuity's Knowledge Base database. Fisher's exact test was then performed to calculate p‐values, and a p‐value < 0.05 was considered statistically significant.

### Statistical Analysis

The statistical analysis of the qPCR and cell count data was performed using GraphPad Prism. OriginPro was used to calculate p‐values using Linear Mixed Models (LMM). We used LMM to account for both fixed and random effects arising due to the differences in the starting cell types in qPCR, as there was non‐independence in the data due to the structure of the study. This further allowed us to prevent noisy results and overestimating the statistical significance. The biological replicates in Figure 3c are separate biological sources listed in Tables S2–S4 (Supporting Information).

### Data Availability

The RNA sequencing data has been submitted to NCBI Gene Expression Omnibus (GEO) and can be accessed under the accession number GSE255748.

## Conflict of Interest

The authors declare no conflict of interest.

## Supporting information

Supporting Information

## Data Availability

The data that support the findings of this study are available from the corresponding author upon reasonable request.
