# Supplementary material for: On‐Chip Neural Induction Boosts Neural Stem Cell Commitment: Toward a Pipeline for iPSC‐Based Therapies
Source: Adv Sci (Weinh). 2024 Apr 24;11(25):2401859. doi: 10.1002/advs.202401859 (PMC11220685; doi:10.1002/advs.202401859)
Supplement: Supplementary file 1 — Supporting Information [file ADVS-11-2401859-s001.pdf]

ADVANCED SCIENCE
Open Access

Supporting Information

for *Adv. Sci.*, DOI 10.1002/advs.202401859

On-Chip Neural Induction Boosts Neural Stem Cell Commitment: Toward a Pipeline for iPSC-Based Therapies

Saumey Jain, Dimitrios Voulgaris, Surangrat Thongkorn, Rick Hesen, Alice Hägg, Mohsen Moslem, Anna Falk and Anna Herland**

Supporting Information

On-chip neural induction boosts human neural stem commitment and stabilization: towards a pipeline for iPSC-based therapies

Saumey Jain, Dimitrios Voulgaris, Surangrat Thongkorn, Rick Hesen, Alice Hägg, Mohsen Moslem, Anna Falk and Anna Herland**

Table S1: Design specifications of the microfluidic chips and the conventional well plates

Cell culture format	Height	Surface area	Volume
Microfluidic chip for reprogramming	0.4 mm	45 mm^2	18 µL
Microfluidic chip for neural induction	0.6 mm	45 mm^2	27 µL
12-well plate	-	350 mm^2	~1000 µL
6-well plate	-	960 mm^2	~2000 µL

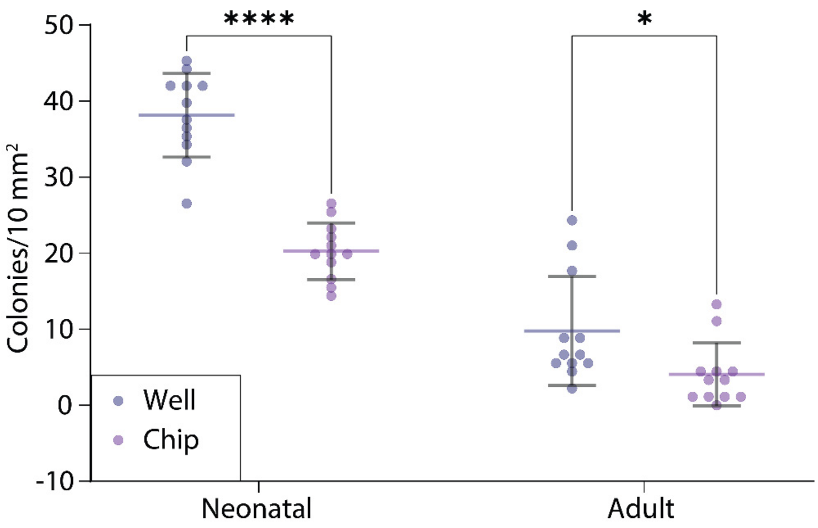

Figure S1: Characterization of iPSCs obtained by reprogramming fibroblasts in the microfluidic format: Number of colonies obtained per 10 mm^2 surface area in the microfluidic devices, with each point on the plot representing data from one image frame.

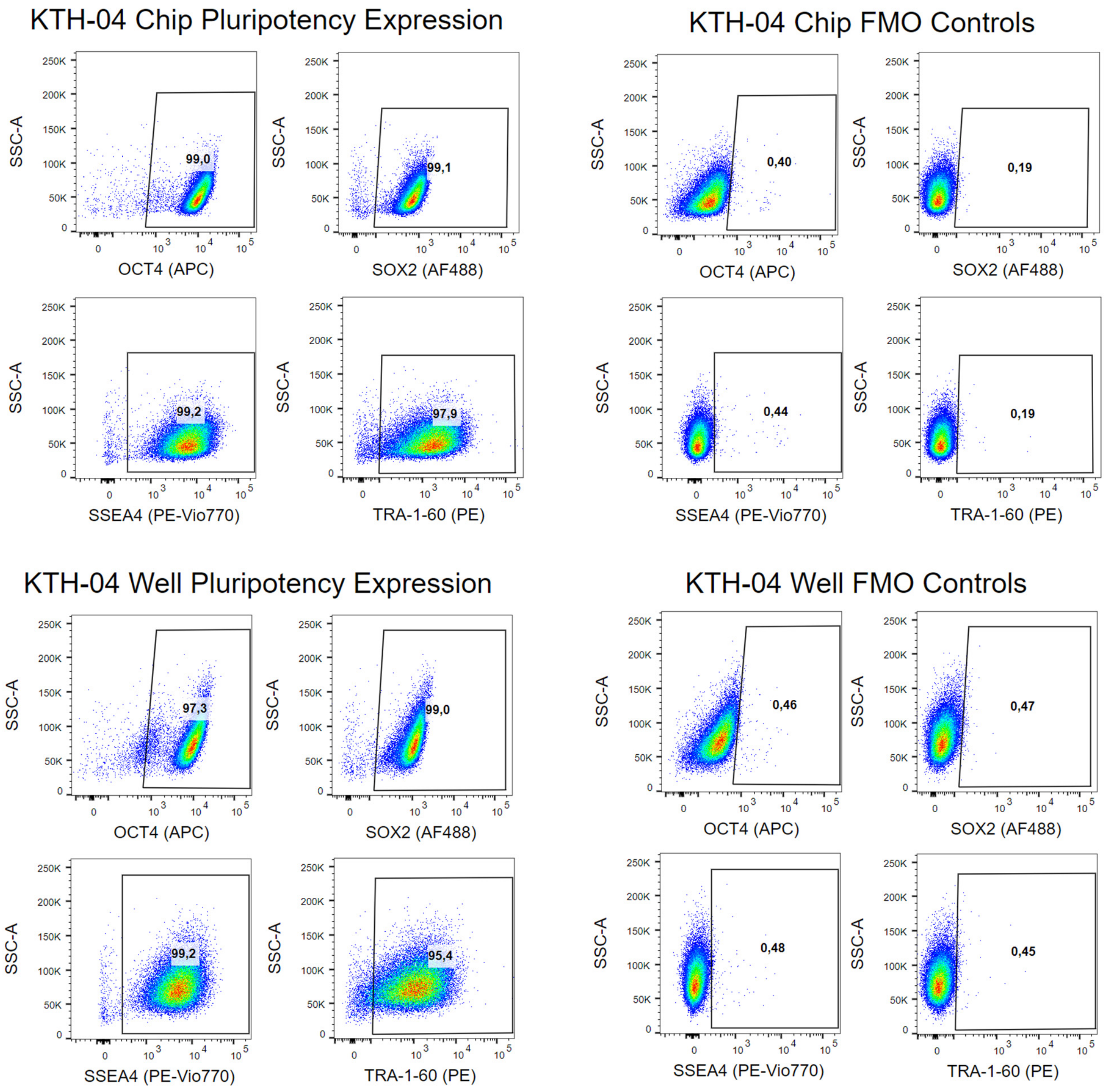
KTH-04 Chip Pluripotency Expression
KTH-04 Chip FMO Controls
KTH-04 Well Pluripotency Expression
KTH-04 Well FMO Controls
SSC-A
OCT4 (APC)
SOX2 (AF488)
SSEA4 (PE-Vio770)
TRA-1-60 (PE)
99,0
99,1
99,2
97,9
0,40
0,19
0,44
0,19
97,3
99,0
99,2
95,4
0,46
0,47
0,48
0,45

KTH-05 Chip Pluripotency Expression

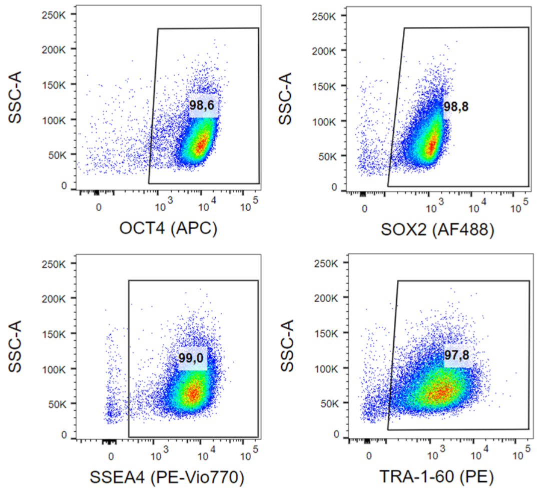

KTH-05 Chip FMO Controls

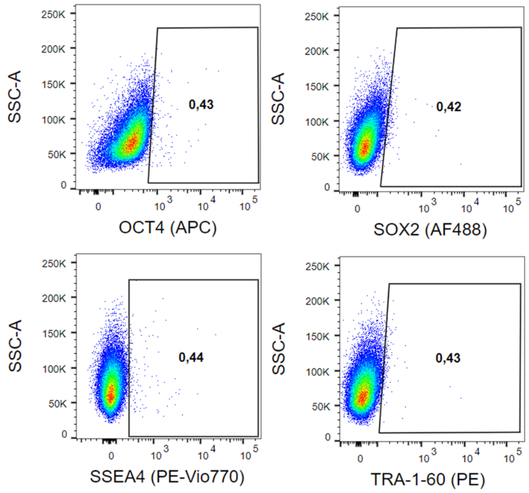

KTH-05 Well Pluripotency Expression

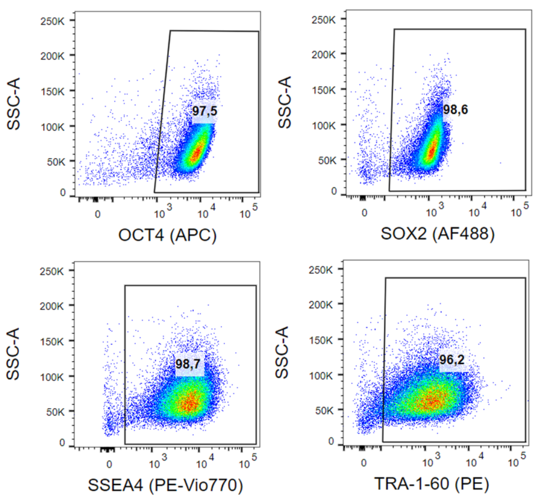

KTH-05 Well FMO Controls

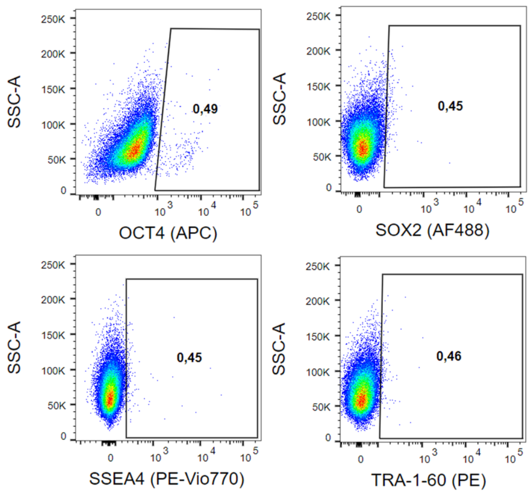

KTH-06 Chip Pluripotency Expression

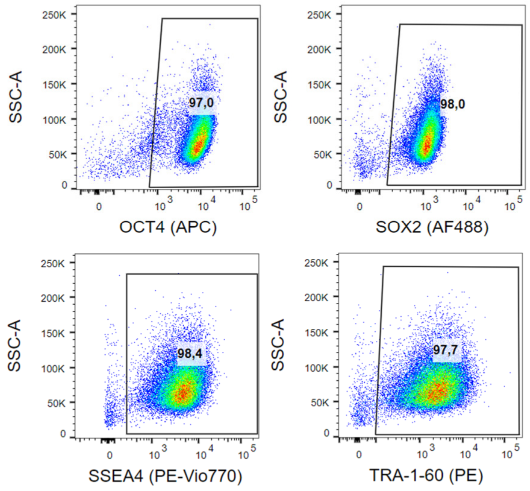

KTH-06 Chip FMO Controls

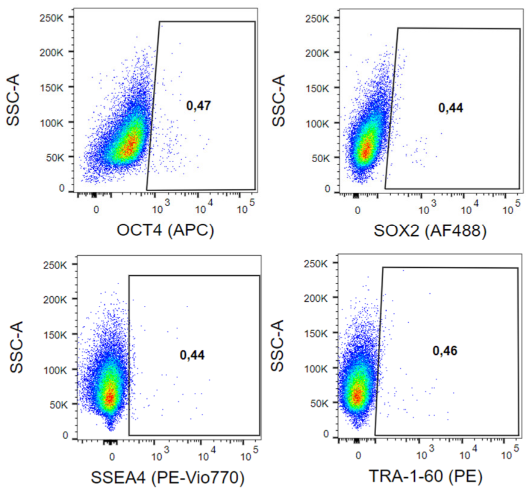

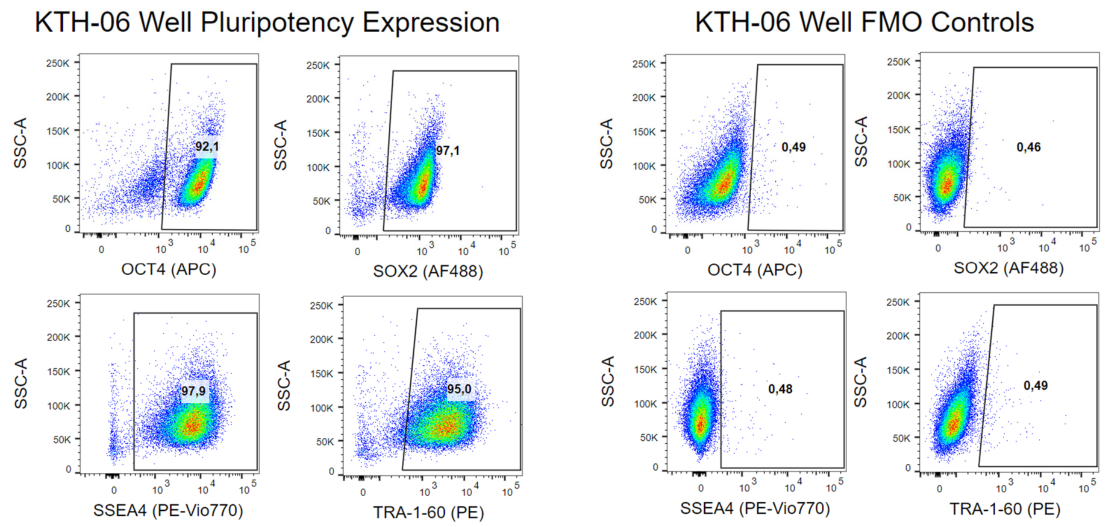

Figure S2: Pluripotency expression in iPSCs generated on chips or in wells. Flow cytometric scatter plots showing pluripotency expression in iPSCs reprogrammed in well or chip, after single cell expansion. KTH-04: well p 19, chip p 20. KTH-05: well p 14, chip p14. KTH-06: well p 14, chip p14. FMO controls were used as negative controls to set the gates on the respective iPSC line co-stained for OCT4, SOX2, SSEA4 and TRA-1-60.

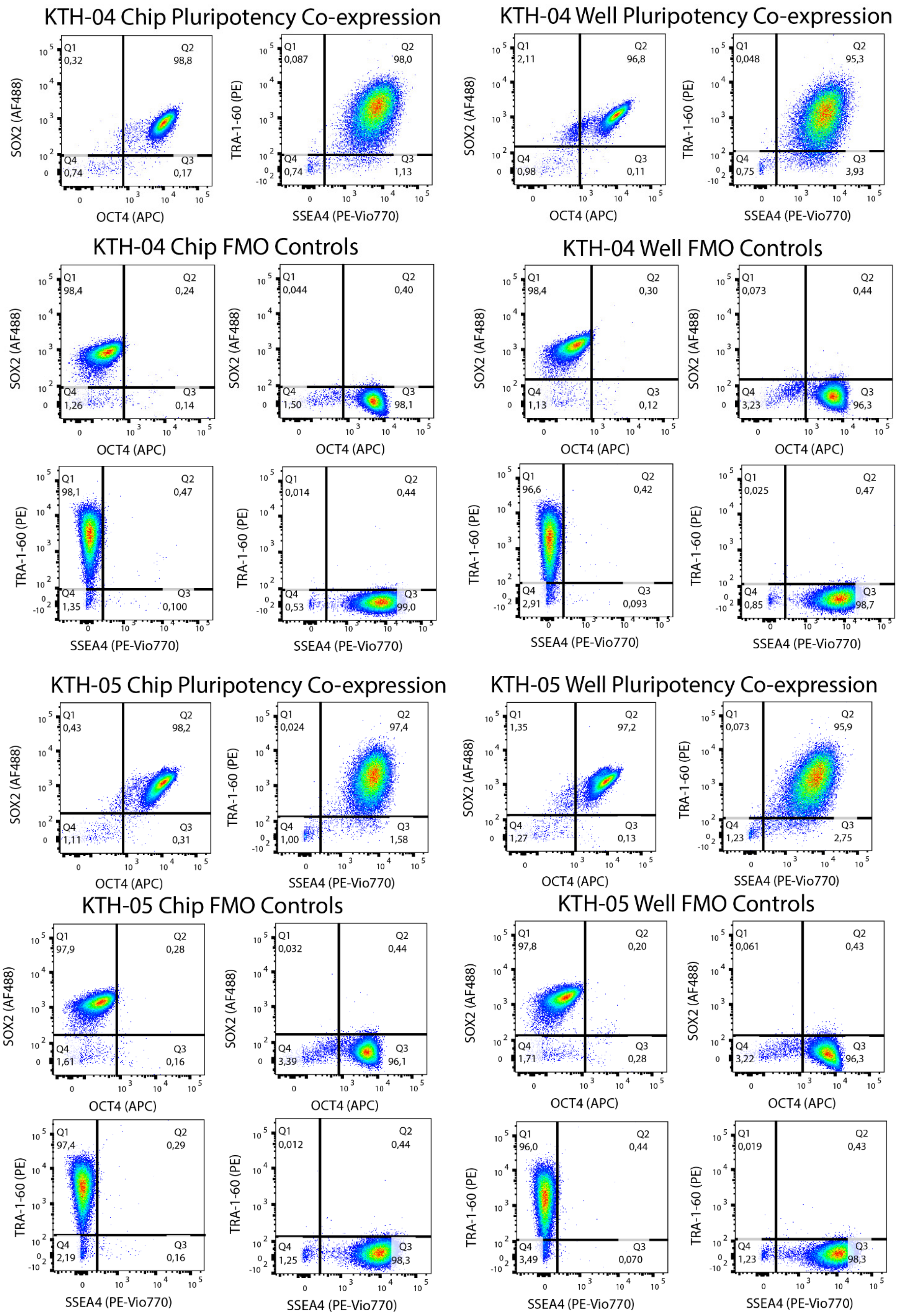
KTH-04 Chip Pluripotency Co-expression
SOX2 (AF488) vs OCT4 (APC): Q1 0,32; Q2 98,8; Q4 0,74; Q3 0,17
TRA-1-60 (PE) vs SSEA4 (PE-Vio770): Q1 0,087; Q2 98,0; Q4 0,74; Q3 1,13
KTH-04 Well Pluripotency Co-expression
SOX2 (AF488) vs OCT4 (APC): Q1 2,11; Q2 96,8; Q4 0,98; Q3 0,11
TRA-1-60 (PE) vs SSEA4 (PE-Vio770): Q1 0,048; Q2 95,3; Q4 0,75; Q3 3,93
KTH-04 Chip FMO Controls
SOX2 (AF488) vs OCT4 (APC): Q1 98,4; Q2 0,24; Q4 1,26; Q3 0,14
SOX2 (AF488) vs OCT4 (APC): Q1 0,044; Q2 0,40; Q4 1,50; Q3 98,1
TRA-1-60 (PE) vs SSEA4 (PE-Vio770): Q1 98,1; Q2 0,47; Q4 1,35; Q3 0,100
TRA-1-60 (PE) vs SSEA4 (PE-Vio770): Q1 0,014; Q2 0,44; Q4 0,53; Q3 99,0
KTH-04 Well FMO Controls
SOX2 (AF488) vs OCT4 (APC): Q1 98,4; Q2 0,30; Q4 1,13; Q3 0,12
SOX2 (AF488) vs OCT4 (APC): Q1 0,073; Q2 0,44; Q4 3,23; Q3 96,3
TRA-1-60 (PE) vs SSEA4 (PE-Vio770): Q1 96,6; Q2 0,42; Q4 2,91; Q3 0,093
TRA-1-60 (PE) vs SSEA4 (PE-Vio770): Q1 0,025; Q2 0,47; Q4 0,85; Q3 98,7
KTH-05 Chip Pluripotency Co-expression
SOX2 (AF488) vs OCT4 (APC): Q1 0,43; Q2 98,2; Q4 1,11; Q3 0,31
TRA-1-60 (PE) vs SSEA4 (PE-Vio770): Q1 0,024; Q2 97,4; Q4 1,00; Q3 1,58
KTH-05 Well Pluripotency Co-expression
SOX2 (AF488) vs OCT4 (APC): Q1 1,35; Q2 97,2; Q4 1,27; Q3 0,13
TRA-1-60 (PE) vs SSEA4 (PE-Vio770): Q1 0,073; Q2 95,9; Q4 1,23; Q3 2,75
KTH-05 Chip FMO Controls
SOX2 (AF488) vs OCT4 (APC): Q1 97,9; Q2 0,28; Q4 1,61; Q3 0,16
SOX2 (AF488) vs OCT4 (APC): Q1 0,032; Q2 0,44; Q4 3,39; Q3 96,1
TRA-1-60 (PE) vs SSEA4 (PE-Vio770): Q1 97,4; Q2 0,29; Q4 2,19; Q3 0,16
TRA-1-60 (PE) vs SSEA4 (PE-Vio770): Q1 0,012; Q2 0,44; Q4 1,25; Q3 98,3
KTH-05 Well FMO Controls
SOX2 (AF488) vs OCT4 (APC): Q1 97,8; Q2 0,20; Q4 1,71; Q3 0,28
SOX2 (AF488) vs OCT4 (APC): Q1 0,061; Q2 0,43; Q4 3,22; Q3 96,3
TRA-1-60 (PE) vs SSEA4 (PE-Vio770): Q1 96,0; Q2 0,44; Q4 3,49; Q3 0,070
TRA-1-60 (PE) vs SSEA4 (PE-Vio770): Q1 0,019; Q2 0,43; Q4 1,23; Q3 98,3

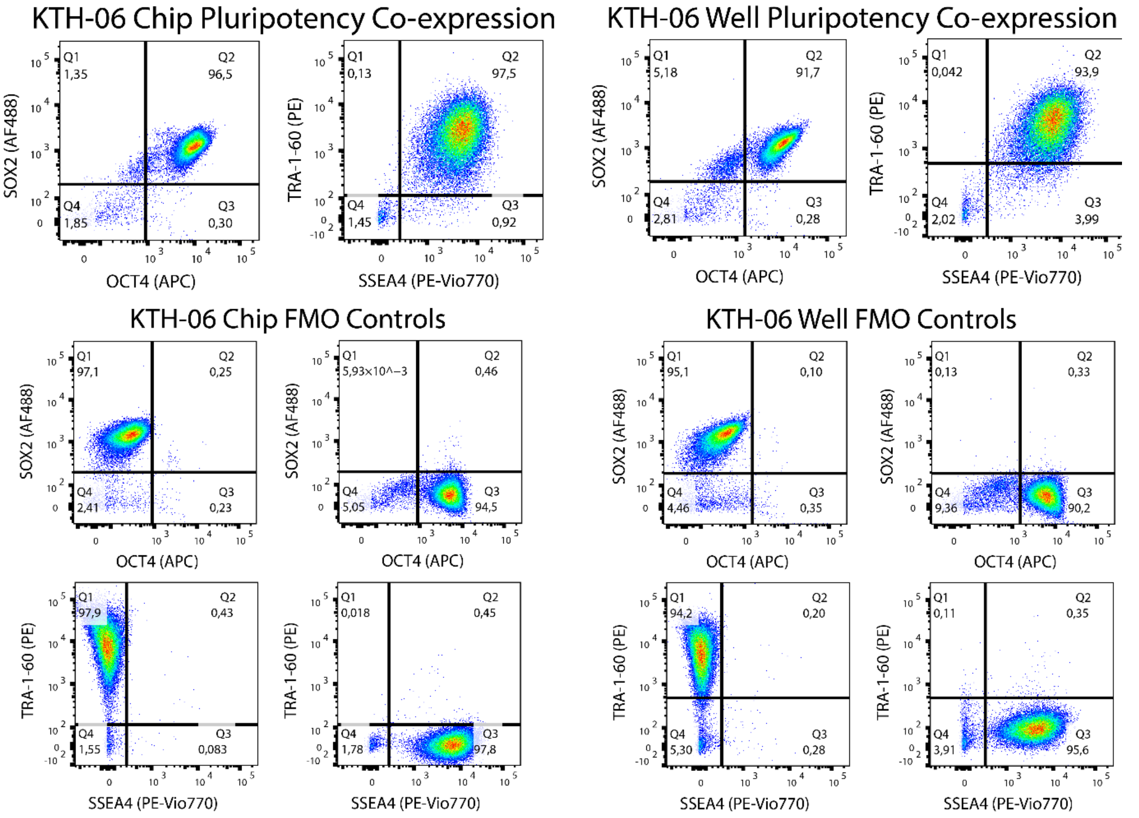

Figure S3: Gating strategies for flow cytometry analysis of pluripotency co-expression in Figure 2c. Flow cytometric scatter plots showing co-expression of intracellular markers OCT4 and SOX2 and extracellular markers SSEA4 and TRA-1-60 in iPSCs reprogrammed in well or chip, respectively. KTH-04: well p 19, chip p 20. KTH-05: well p 14, chip p14. KTH-06: well p 14, chip p14. Cell line-specific FMO controls were used as negative controls to set the gates.

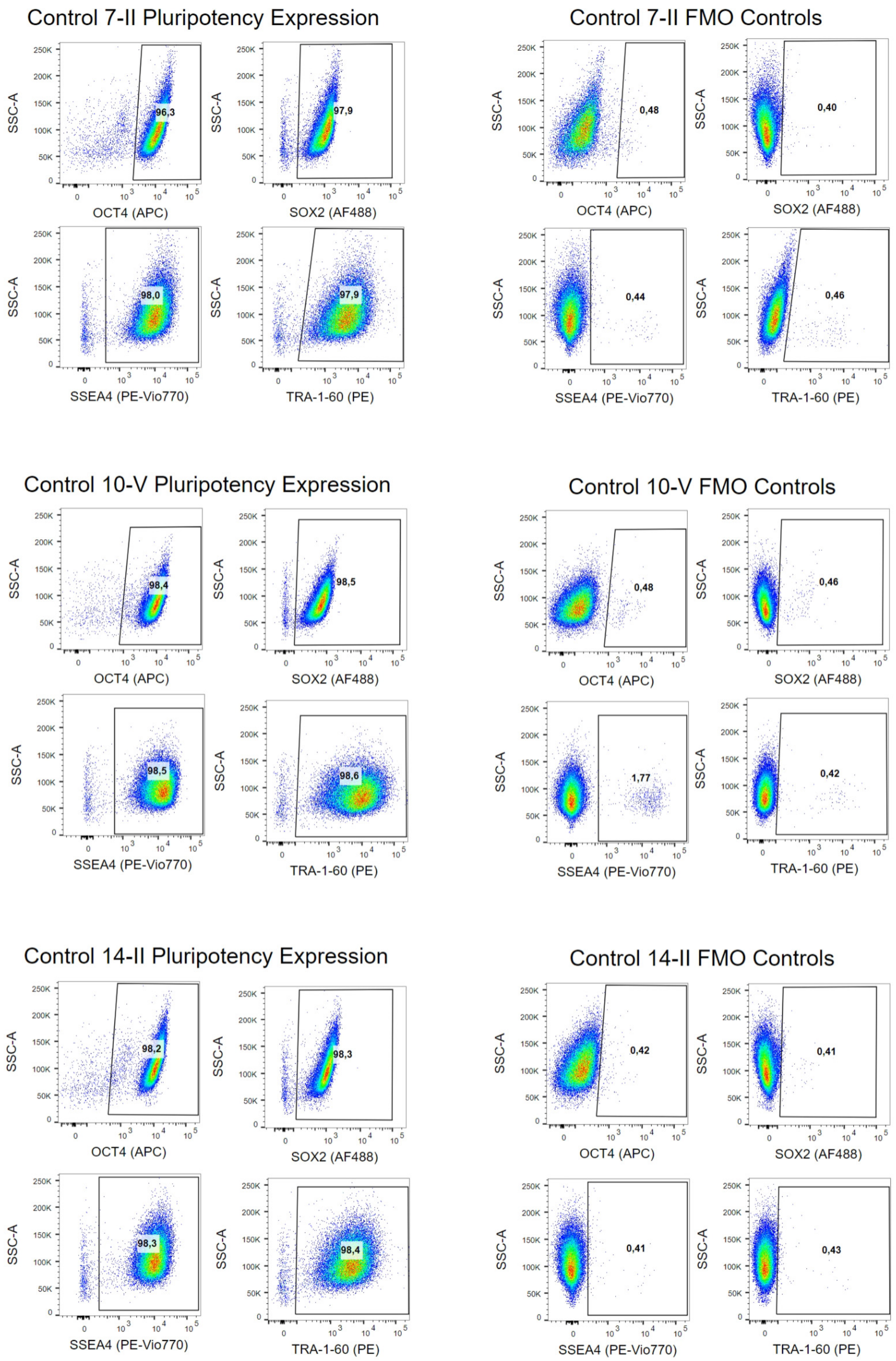

Figure S4: Pluripotency expression in clonal iPSC lines. Flow cytometric scatter plots showing pluripotency expression in iPSC lines obtained from the iPS Core at Karolinska Institute: Control 7-II p 18, Control 10-V p 33, Control 14-II p 38. FMO controls were used as negative controls to set the gates on the respective iPSC line co-stained for OCT4, SOX2, SSEA4, and TRA-1-60.

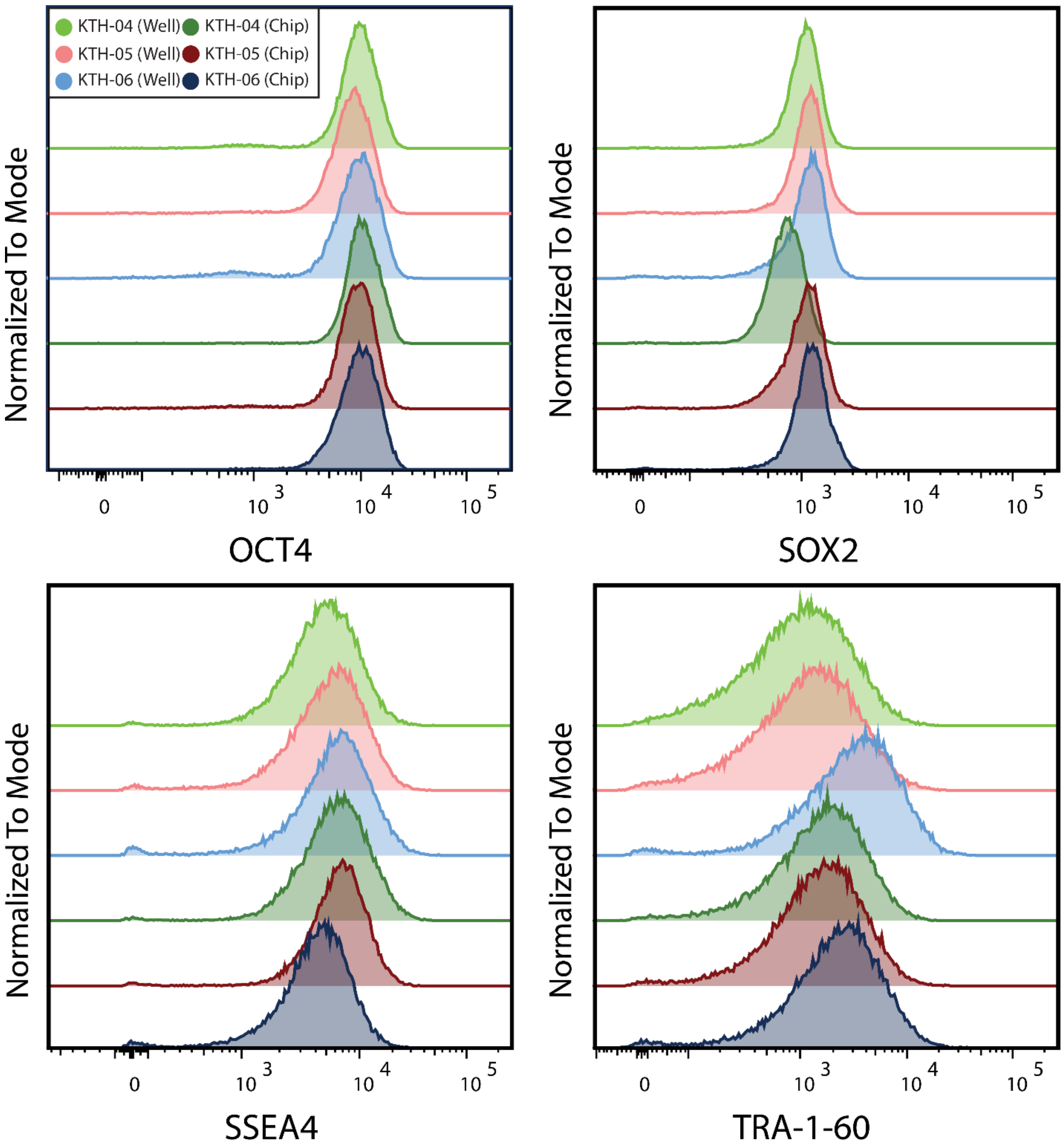

Figure S5: An overview of the intensity of expression for OCT4, SOX2, SSEA4, and TRA-1-60 in iPSCs reprogrammed in well or chip after single cell expansion. Quantitative expression data are shown in flow cytometric scatter plots in Figure S4. This used the following cell lines KTH-04: well p 19, chip p 20. KTH-05: well p 14, chip p14. KTH-06: well p 14, chip p 14.

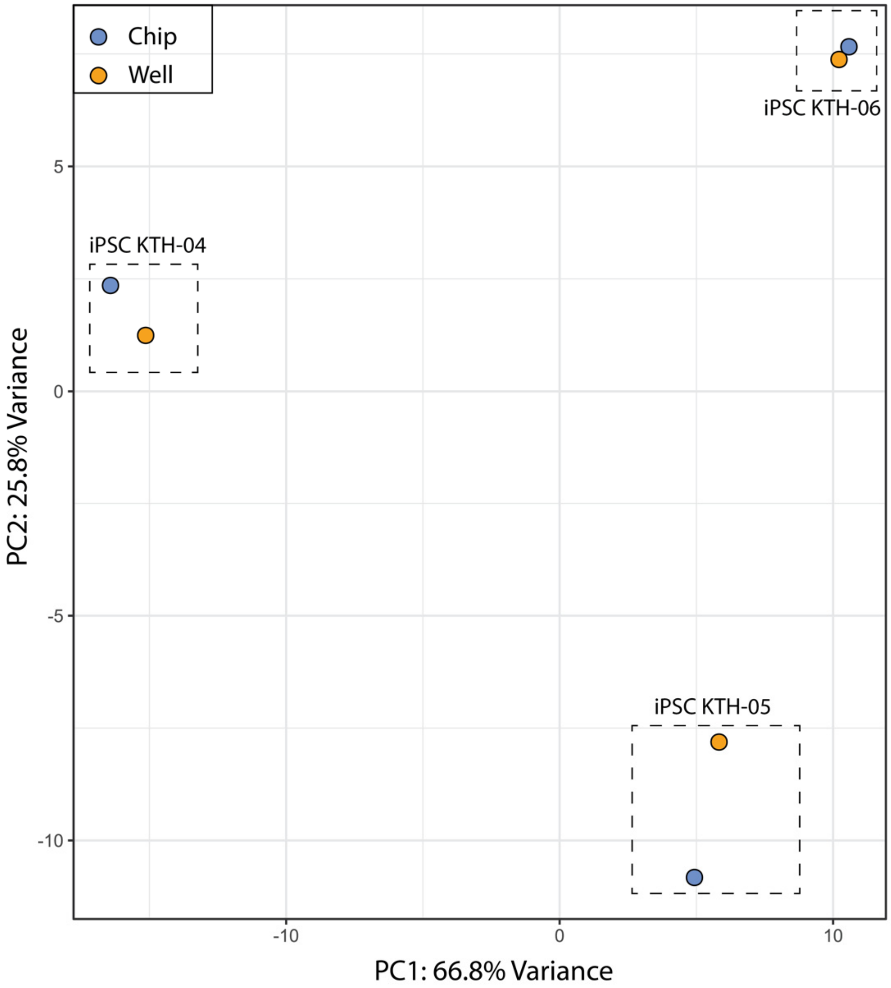

Figure S6: PCA of iPSCs obtained by reprogramming in a conventional well plate and microfluidic platforms. iPSCs obtained from both conditions usually cluster close to each other, highlighting no substantial difference between the cells obtained.

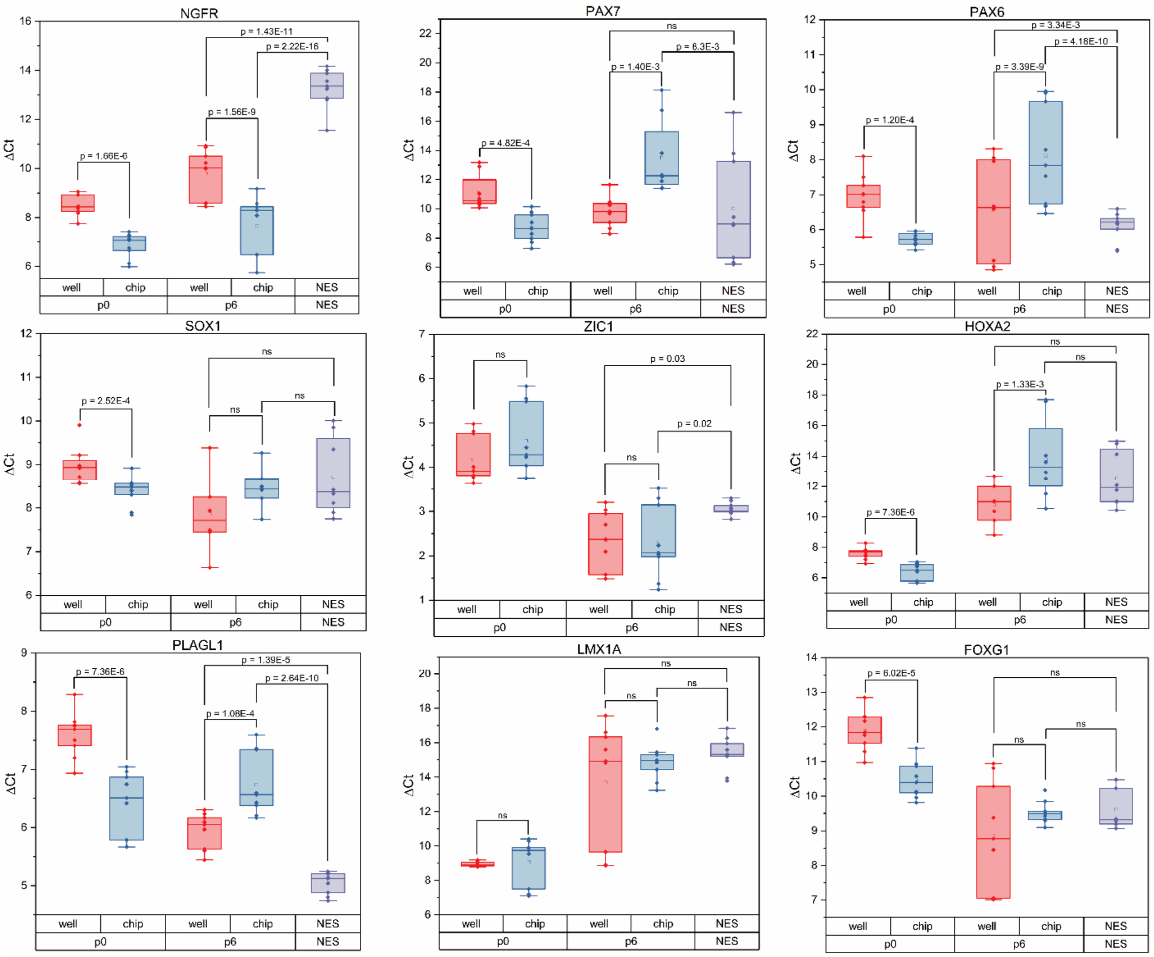

Figure S7: Statistical analysis of the protein expression data obtained by qPCR on neural differentiated cells on both well and chip culture formats. The p-values were determined using the Linear Mixed Model on OriginPro 2023b. There were three biological replicates analyzed for each data point, and each point represents one technical replicate. The biological replicates are listed in Table S9, Table S10 and Table S11. The error bars signify the standard deviation, the centerline signifies the median value, the square signifies the mean, and the box plots correspond to the 25 and 75 percentiles.

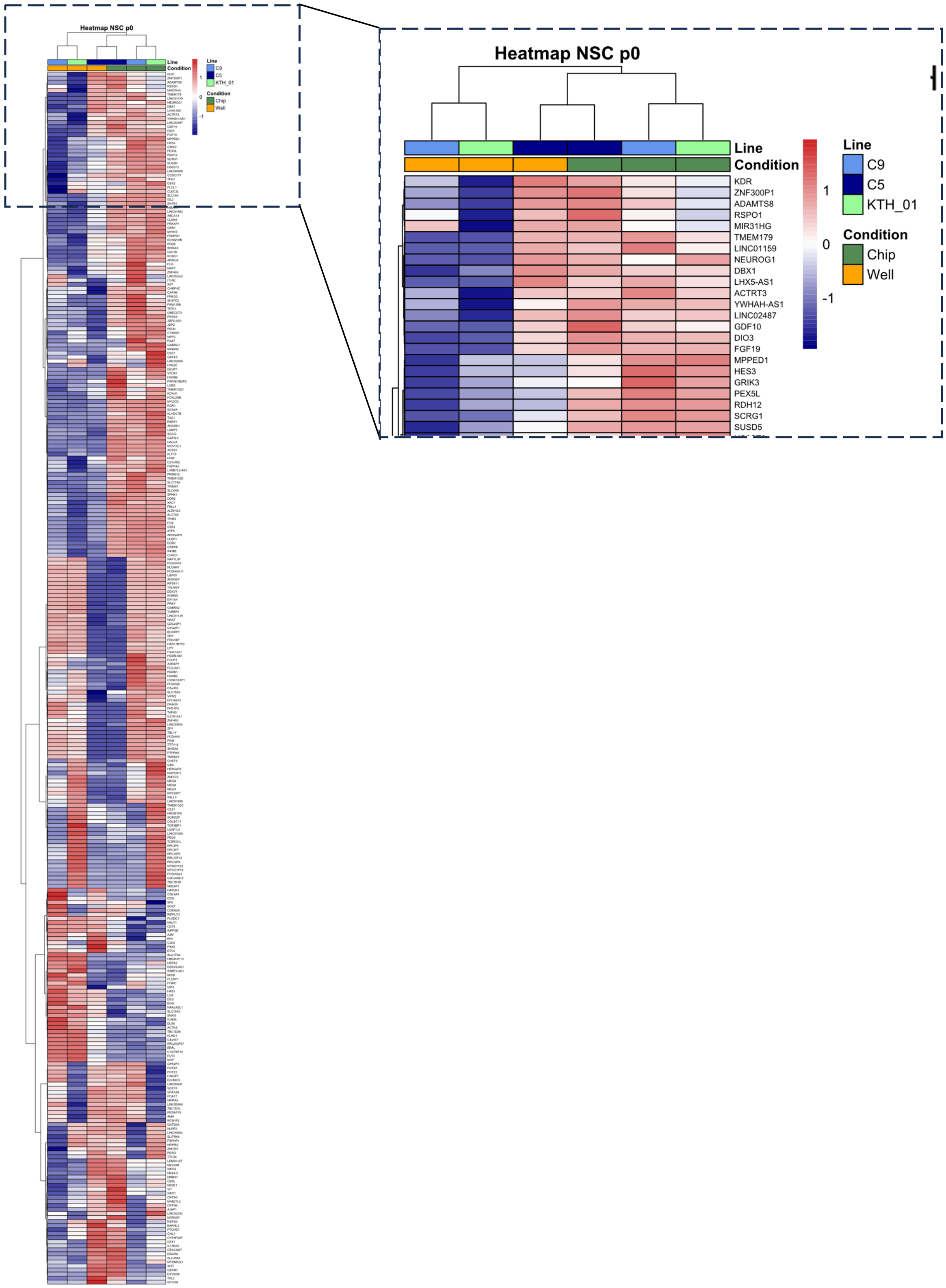

Figure S8: Hierarchical clustering of NSC for the time point "generation" for the 300 most variable genes. NSCs generated on chips (yellow) clustered together; the same pattern was evident for NSCs generated in wells (blue). The deviation of C5 is also evident here, where the chip and well conditions are associated more with each other than with the chip and well conditions of C9 and KTH-01. The figure inset provides a better view of the hierarchical clustering observed.

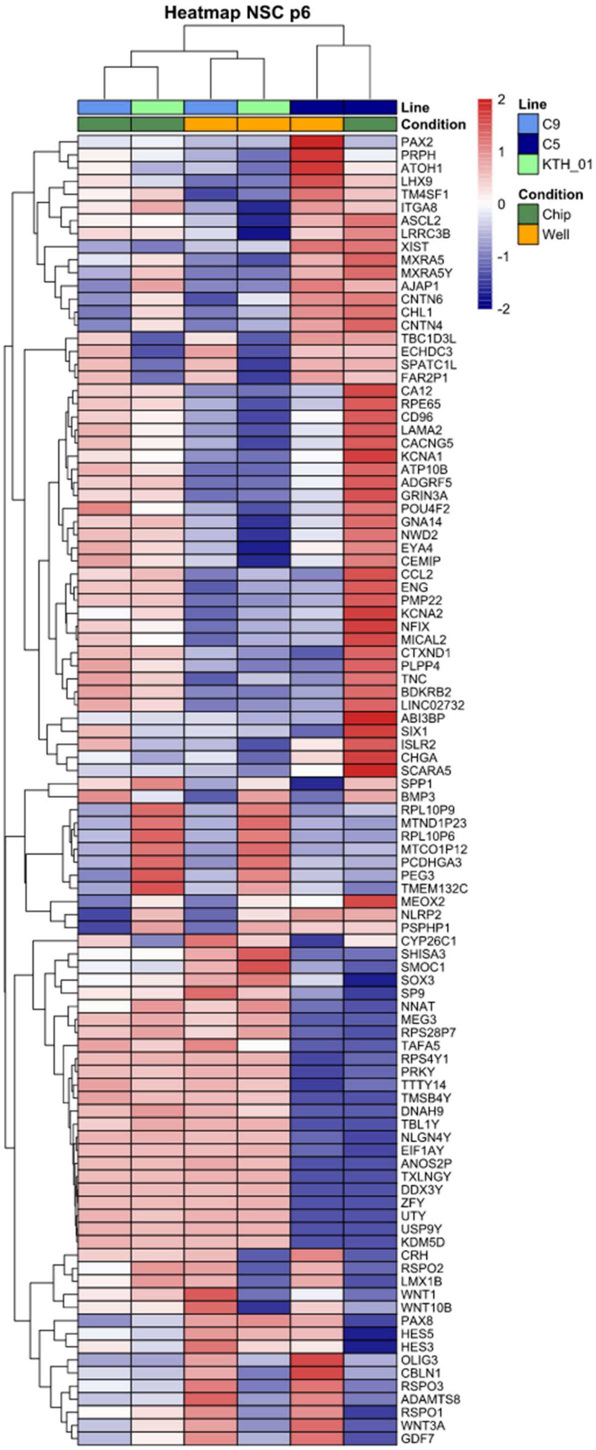

Figure S9: Hierarchical clustering of NSC for the time point "maintenance" for the most variable genes. NSCs generated on chips (yellow) clustered together; the same pattern was evident for NSCs generated in wells (blue). The deviation of C5 is also evident here, where the chip and well conditions are associated more with each other than with the chip and well conditions of C9 and KTH-01.

Table S2: Summary of cell sources and iPSC lines generated

Cell Source	Vendor	Cat #	Line generated	Sex	Age	Ethnicity or skin pigmentation
Fibroblast 1	Thermo Scientific	C0045C	iPS KTH-(01-04)	M	Neonatal	Black
Fibroblasts 2	ATCC	PCS-201-012	iPS KTH-05	F	34	White
Fibroblasts 3	Cell Applications, Inc.	106-05a	iPS KTH-06	F	41	White

Table S3: Summary of cell sources and NSC lines generated

Cell Source	Reprogramming technique	Line generated	Sex	Age
KTH-01	mRNA	NSC KTH-01	M	Neonatal
CTRL-09	mRNA/Sendai Virus	NSC CTRL-09	M	Child
CTRL-05	Sendai Virus	NSC CTRL-05	F	Child

Table S4: Summary of reference lines for reprogramming and neural induction

iPS Cell Line	Reprogramming technique	NES cell line	Sex	Age
iPSC CTRL-14	mRNA	NES CTRL-14 p.33	F	Child
iPSC CTRL-09	mRNA/Sendai Virus	NES CTRL-09 p.21	M	Child
iPSC KICR001-A	mRNA	NES KICR001-A p.20	M	Old

Table S5: Media for Neural Induction

Media	Basal Media	ß-mercaptoethanol	NEAA	N2	B27
Knock our serum replacement (KOSR)	DMEM:F12 + Glutamax, 20% KOSR	90 µM	1x	-	-
N2B27	DMEM:F12 + Glutamax : Neurobasal (1:1)	90 µM	-	1:200	1:100

Table S6: Media for Neural Induction

<table>
<tr><th>Day</th><th>KOSR/N2B27</th><th>Noggin</th><th>SB431542</th><th>CHIR99021</th></tr>
<tr><td>0-3</td><td>100% KOSR</td><td rowspan="4">500 ng/mL</td><td>10 µM</td><td rowspan="5">3.33 µM</td></tr>
<tr><td>4-5</td><td>75% / 25%</td><td rowspan="4">-</td></tr>
<tr><td>6-7</td><td>50% / 50%</td></tr>
<tr><td>8-9</td><td>25% / 75%</td></tr>
<tr><td>10-11</td><td>100% N2B27</td><td>-</td></tr>
</table>

Table S7: TaqMan Probes used for qPCR

TaqMan Gene Expression assay	Assay ID	Gene Description
COL1A1-FAM	Hs00164004_m1	Fibroblast marker
VIM-FAM	Hs00958111_m1	Mesenchymal marker
CD44-FAM	Hs01075864_m1	Adhesion associated marker
Pou5F1-FAM (OCT4)	Hs00999632_g1	iPSC pluripotency marker
SOX2-FAM	Hs01053049_s1	iPSC pluripotency marker
NANOG-FAM	Hs02387400_g1	iPSC pluripotency marker
ZIC1-FAM	Hs00602749_m1	Neuroepithelial stem cell marker
SOX1-FAM	Hs01057642_s1	Neuroectoderm marker
PLAGL1-FAM	Hs00414677_m1	Neuroepithelial stem cell marker
PAX6-FAM	Hs01088114_m1	Neuroepithelial stem cell marker
FOXG1-FAM	Hs01850784_s1	Regionality forebrain marker
GBX2-FAM	Hs00230965_m1	Regionality hindbrain marker
NGFR-FAM	Hs00609976_m1	Neural crest cell marker
GAPDH-VIC	Hs0275899_g1	Reference gene marker
